# Artificial intelligence in advanced endoscopic imaging: transforming optical diagnosis in gastroenterology

**DOI:** 10.3389/fmed.2025.1719145

**Published:** 2026-01-16

**Authors:** Sarah Bencardino, Ilaria Lodola, Lucia Centanni, Francesco Vito Mandarino, Jacopo Fanizza, Federica Furfaro, Ferdinando D’Amico, Lorenzo Fuccio, Angelo Bruni, Antonio Facciorusso, Sara Massironi, Vito Annese, Silvio Danese, Andrew A. Gumbs, Gianfranco Donatelli, Giuseppe Dell’Anna

**Affiliations:** 1 Gastroenterology and Gastrointestinal Endoscopy Unit, IRCCS San Raffaele Hospital, Milan, Italy; 2 Faculty of Medicine and Surgery, Vita-Salute San Raffaele University, Milan, Italy; 3 Unit of Gastroenterology, Department of Medical and Surgical Sciences, S. Orsola-Malpighi University Hospital, University of Bologna, Bologna, Italy; 4 Faculty of Medicine, Gastroenterology Unit, University of Salento, Lecce, Italy; 5 Gastroenterology and Gastrointestinal Endoscopy Unit, IRCCS Policlinico San Donato, San Donato Milanese, Italy; 6 Department of Digestive Minimally Invasive Surgery, Antoine Béclère Hospital, Paris, France; 7 Department of General, Visceral, Vascular and Transplant Surgery, University Hospital Magdeburg, Magdeburg, Germany; 8 Unité d’Endoscopie Interventionnelle, Hôpital Privé des Peupliers, Ramsay Générale de Santé, Paris, France; 9 Department of Clinical Medicine and Surgery, University of Naples “Federico II”, Naples, Italy

**Keywords:** adenomas, artificial intelligence, Barrett’s esophagus, early gastric cancer, endoscopic imaging, inflammatory bowel disease, optical biopsy

## Abstract

The term Artificial intelligence (AI) is revolutionizing gastrointestinal (GI) endoscopy by enhancing advanced imaging techniques such as Narrow Band Imaging (NBI), Linked Color Imaging (LCI), iSCAN, and Confocal Laser Endomicroscopy (CLE). AI-driven deep learning algorithms, particularly convolutional neural networks (CNNs) and transformer-based models, have demonstrated high accuracy in the real-time detection, classification, and risk stratification of premalignant and malignant lesions, thereby reducing unnecessary biopsies and improving diagnostic efficiency. In the upper GI tract, AI has shown superior performance in detecting dysplasia in Barrett’s esophagus, distinguishing early gastric cancer from benign alterations, and predicting submucosal invasion depth. This capability enhances decision-making regarding endoscopic resection, such as endoscopic submucosal dissection (ESD). In the lower GI tract, AI is increasingly applied for real-time identification of adenomas, serrated lesions, and neoplastic changes in ulcerative colitis. Studies have confirmed that AI-assisted colonoscopy significantly increases adenoma detection rates, thereby reducing the incidence of interval colorectal cancer. Furthermore, AI-powered advanced endoscopy allows for a more objective assessment of mucosal and histological healing in IBD, helping predict outcomes and advancing precision medicine in its management. This narrative review comprehensively analyzes AI’s role in advanced endoscopic imaging, highlighting its impact on optical diagnosis in both upper and lower GI pathologies. It explores the integration of multimodal AI approaches, which combine imaging data with clinical and molecular biomarkers, to enhance diagnostic precision. Additionally, it discusses current challenges, including the need for multicenter validation, standardization of AI algorithms, and ethical considerations for clinical implementation. Future perspectives emphasize the necessity for high-quality prospective studies to validate AI’s real-world applicability and long-term benefits in endoscopic practice.

## 1 Introduction

Gastrointestinal endoscopy is a cornerstone of diagnostic and therapeutic practice in gastroenterology, enabling direct visualization of mucosal pathology and real-time intervention. Despite major advances in optics and imaging, diagnostic performance remains limited by operator dependency, subjective interpretation, and variability in lesion recognition. These limitations highlight an ongoing need for objective, standardized approaches that can enhance accuracy and reproducibility in endoscopic diagnosis. Integrating artificial intelligence (AI) offers promising support in the form of enhanced lesion detection, improved workflow and more personalized approaches. AI systems can process large volumes of endoscopic data and incorporate patient-specific variables, including genetic information, to support tailored clinical decision-making (1).

Over the past decades, GI endoscopy has evolved through several major technological transitions. The shift from fiber-optic scopes to charge-coupled device (CCD) video endoscopes provided higher-resolution white-light images but still required dye-based chromoendoscopy to highlight subtle mucosal changes (2, 3). This technique, though effective, was labor-intensive and not widely adopted.

The introduction of Narrow-Band Imaging (NBI) represented a major step forward, using filtered illumination at specific wavelengths (415 nm blue and 540 nm green) strongly absorbed by hemoglobin to enhance visualization of superficial vasculature and mucosal microstructure (2, 4, 5). NBI has demonstrated superior performance to conventional white-light endoscopy (WLE) in detecting early neoplasia across several GI organs and has facilitated the development of standardized classification systems (2, 6).

Subsequent advances have focused on software-based virtual chromoendoscopy. i-SCAN applies real-time digital algorithms to enhance surface, contrast and tone, enabling per-pixel adjustment of sharpness and color without altering the light source, improving border delineation and lesion detection (2, 3, 5).

Similarly, Fujifilm developed Linked Color Imaging (LCI). This technology combines pre- and post-processing to produce bright, high-contrast images increasing adenoma detection rates and improving the recognition of early gastric cancer (7–9).

Confocal Laser Endomicroscopy (CLE) introduced the concept of an *in vivo* “optical biopsy,” providing real-time cellular-level imaging when combined with fluorescent dyes (10). Consensus statements and randomized studies have demonstrated that adding pCLE (probe-based) to standard protocols increases the detection of Barrett’s neoplasia and other GI dysplasia (10, 11). Although CLE offers high diagnostic accuracy, its application is limited by cost, narrow field of view, and the need for specific training (5, 10).

Taken together, the evolution from optical filters (NBI) and digital post-processing (i-SCAN) to enhanced color contrast (LCI) and intramucosal microscopy (CLE) illustrates a clear shift toward real-time, minimally invasive optical diagnosis. This evolution improves mucosal visualization and microvascular detail provided that operators receive appropriate training and use validated classification schemes (2).

Despite substantial technological progress in GI endoscopy, current optical diagnostic approaches still have significant limitations in terms of variability between operators and across centers, leading to inconsistent diagnostic accuracy and persistent reliance on histopathologic confirmation (12).

AI has emerged as a promising solution to address these unmet needs. Deep learning (DL) models, particularly convolutional neural networks (CNNs), can process large volumes of endoscopic images and video data to automatically detect, localize, and characterize mucosal abnormalities. These systems have already shown strong performance in real-time detection of colorectal polyps, early colorectal cancer, and esophageal neoplasia, often matching or exceeding expert-level accuracy. Beyond detection, AI is being developed to assist in lesion characterization, automated report generation, and workflow optimization. These systems could reduce human variability and offer the potential to stratify lesions more reliably during the procedure, thus supporting more selective and evidence-based biopsy decisions (13).

Early AI systems in endoscopy operate through two complementary components: computer-aided detection (CADe), which identifies and highlights suspicious areas of mucosa in real time, and computer-aided diagnosis (CADx), which characterizes detected lesions and predicts their histological nature, such as distinguishing neoplastic from non-neoplastic tissue (1, 14). With the integration of DL and CNNs, AI models now autonomously learn from large volumes of endoscopic images, recognizing subtle patterns and mimicking the structure of the human visual cortex. These models are capable of real-time image interpretation, offering enhanced accuracy in both detection and classification of lesions across various GI regions (1, 15).

Nevertheless, the field faces practical hurdles, such as limited guidelines for everyday use, lack of reimbursement and concerns for over-diagnosis, highlighting the need for balanced implementation and further prospective validation (15, 16).

AI is increasingly facilitating the integration of diverse data sources, including endoscopic imaging, histologic slides, clinical variables, and molecular biomarkers, into unified predictive models, paving the way for a more personalized approach to gastroenterology (17). In the context of IBD, this integrative “endo-histo-omic” strategy shows remarkable potential, with AI models successfully merging high-definition endoscopic features and transcriptomic signatures to anticipate response to biologic treatment and forecast long-term disease trajectories (17, 18).

Beyond IBD, technological progress has led to the development of multimodal imaging systems, capable of simultaneously capture optical coherence tomography (OCT), fluorescence, and photoacoustic signals within a single acquisition. This creates co-registered image datasets that incorporate structural, functional, and biochemical information. Such synchronized inputs are ideal for training deep-learning models to correlate endoscopic optical features with molecular risk markers across a variety of GI lesions (19).

AI tools have also demonstrated high sensitivity in detecting early-stage cancers by analyzing specific image features such as size, shape, and color. These systems also potentially improve polyp detection rates and enhance real-time classification of polyps, throught heatmaps or boxes around polyps. When these capabilities are integrated into multimodal pipelines, combining visual insights and molecular data, they offer further diagnostic precision (20). Furthermore, capsule endoscopy enhanced by AI can now incorporate laboratory and clinical data to detect hidden bleeding sources with high sensitivity, enabling more targeted device-assisted enteroscopy and reducing diagnostic delays (1).

The integration of AI with advanced optical technologies such as NBI, i-SCAN, LCI, and CLE could enable multimodal AI platforms capable of providing comprehensive, real-time assessment. These systems could combine visual, textural, and even patient-specific clinical data to deliver more accurate, reproducible, and personalized endoscopic diagnoses.

In summary, the evolution of GI endoscopy reflects a steady movement from macroscopic inspection toward high-resolution, real-time optical diagnosis. Yet, despite technological sophistication, human interpretation remains the main limiting factor. The incorporation of AI represents the next paradigm shift, transforming endoscopy into a data-driven, standardized, and intelligent diagnostic modality. This review will explore how AI is being integrated into advanced endoscopic imaging, the current challenges in optical diagnosis, and the potential of multimodal AI systems to improve diagnostic accuracy, reduce reliance on histology, and support precision endoscopic care.

## 2 AI in advanced endoscopic imaging for upper GI

### 2.1 Barrett’s esophagus and esophageal adenocarcinoma

Barrett’s esophagus (BE), which is defined as a columnar-lined esophagus extending proximally by at least 1 cm with specialized intestinal metaplasia in biopsy samples (21), is the only identifiable precursor of esophageal adenocarcinoma (EAC). Progression occurs through a dysplastic sequence, from low-grade dysplasia (LGD) to high-grade dysplasia (HGD) and invasive cancer (22). Identifying early neoplastic changes in BE remains challenging, as these lesions often present with subtle mucosal and vascular alterations. Although modern high-definition endoscopes can reveal even the most subtle abnormalities, the main limitation of early diagnosis currently lies in the endoscopist’s ability to accurately recognize and interpret these early signs of neoplasia. A meta-analysis found that approximately 20% of HGD or EAC on BE were detected within 1 year of the initial diagnosis suggesting that these lesions had likely been missed at the time of the initial diagnosis and highlighting the importance of performing high-quality endoscopy (23).

CADe systems could support endoscopists in detecting these precancerous lesions, thereby facilitating timely endoscopic therapy, such as endoscopic mucosal resection (EMR) and radiofrequency ablation (RFA), and reducing the need for invasive surgery (24).

#### 2.1.1 AI-assisted detection of dysplasia and early neoplasia

Several CADe systems have been developed for Barrett’s esophagus in recent years.

One of the most rigorously trained AI systems for BE-related neoplasia is the deep learning-based CADe model developed by De Groof e al. (25).

This model was trained using a large, multi-institutional dataset comprising 1,704 high resolution endoscopic images, including BE related neoplasia (BERN) and non-dysplastic BE (NDBE), annotated by over 10 expert endoscopists with histopathological confirmation. To improve generalizability and minimize overfitting, the system underwent both internal and double external validation. When tested on external datasets, the CADe system achieved an accuracy of 88%, a sensitivity of 93% and a specificity of 83%. This performance surpassed that of thereby outperforming non-expert endoscopists. Furthermore, the algorithm correctly identified the optimal biopsy site in over 90% of cases.

A further step was the application of AI algorithms to images captured in real time during endoscopic procedures, with the aim of evaluating the potential to support clinical decision making. This approach was first explored by De Groof’ group, who applied their CADe system to still images acquired intra-procedurally in a pilot study involving 20 patients (10 with BE dysplasia and 10 with NDBE) demonstrating the feasibility of *in vivo* application (26, 27). This was subsequently improved upon by Ebigbo and colleagues who developed a faster algorithm and applied it to a larger dataset, achieving excellent performance (sensitivity 83.7%, specificity 100% and accuracy 89.9%) and strong agreement with expert endoscopists.

This AI system required the endoscopist to manually freeze the live endoscopy video before analysis. This reliance on endoscopist input may limit the system’s generalizability beyond expert centers (28).

Despite promising results, all currently available AI systems for BE-related neoplasia share a fundamental limitation: they rely on still images. This introduces a considerable selection bias, as only well-framed, high-quality or suspicious areas are captured. Overcoming this constraint will require AI models trained on full motion endoscopic videos which better reflects the complexity of clinical practice.

This limitation was first addressed by Abdelrahim and colleagues, who developed and validated a deep learning model on both images and full motion pre-recorded endoscopic videos from four different European centers and across all three major endoscopy platforms (Fujifilm, Olympus, Pentax). The system achieved an overall accuracy of 92%, with a sensitivity of 93.8% and a specificity of 90.7%, outperforming endoscopist’s performance (63.5, 77.9, and 71.8%, respectively) (29). Hussein and colleagues expanded this line of work by developing a system capable of delineate the exact borders of dysplastic lesions—an inherently difficult task due to substantial interobserver variability even among experts. While the model showed limited performance in precise boundary segmentation, as reflected by a mean Dice coefficient of 0.5, it consistently identified the lesion’s most suspicious region, offering valuable guidance for target biopsies (30–32).

Building on these foundations, Fockens et al. (33) conducted a large multicentre study on a CADe system using a substantially larger and prospectively collected dataset from 15 centers. Like the previous studies, the system was trained and tested on prerecorded full motion endoscopic videos. However, recording was obtained without prior focus on the neoplastic areas, thereby approximating routine surveillance conditions. Importantly, the CADe system significantly improved general endoscopists’ sensitivity for neoplasia detection from 74 to 88% for still images and from 67 to 79% for videos. A distinctive strength of this study was the inclusion of both general and expert endoscopists in comparative analysis. A key limitation, however, was that the system was tested using only Olympus endoscopic equipment, which may affect generalizability to other platforms.

All of these studies are trained on high-quality, expert-acquired datasets that do not reflect the heterogeneous conditions encountered in routine clinical practice. This discrepancy leads to a phenomenon known as the “domain gap”—a situation where models perform well on familiar, high-quality data, but show reduced accuracy when applied to data that falls outside their training distribution such as lower-quality images. Ideally, the test dataset should be sourced from a wide range of community centers to fully capture the heterogeneity, including differences in endoscopist expertise and sedation methods (34).

Unfortunately, these studies do not fully capture how endoscopists integrate AI into their decision making progress—often shaped by varying levels of skepticism toward AI systems and automation bias (35).

Moreover, this complex human-machine interaction is further complicated by the fact that most AI tools have been primarily validated for the detection of HGD and early esophageal adenocarcinoma (EAC). Applying these tools to the detection of low-grade dysplasia—more commonly targeted in surveillance programs—can introduce bias in real-world settings, underscoring the need for a more cautious and context-aware clinical approach. A evaluation of endoscopist-CADe interaction is therefore crucial to assess the real benefit of these CADe systems (36).

A new architecture gaining attention in the field of image analysis is the Vision Transformer (ViT), which offers an alternative to conventional convolutional approaches by relying on self-attention mechanisms. A recent study applied such models to the detection of Barrett’s neoplasia using still endoscopic images, achieving a sensitivity of 94%—among the highest reported. Importantly, the study introduced a multi-modality training approach, combining white light imaging (WLI) and narrow band imaging (NBI) outperforming single-modality versions. While this concept had been previously explored by Hashimoto et al. (37), the new study expanded on it with a larger dataset and a more robust methodology.

#### 2.1.2 From detection to decision: AI for diagnostic and therapeutic stratification in BE

The detection of early neoplasia in BE typically involves a two-step process: a broad inspection performed using white-light endoscopy (WLE) to identify mucosal abnormalities and then a more targeted evaluation of suspicious areas with optical chromoendoscopy. Within this diagnostic workflow, CADx systems can support lesion characterization, aid in the resection margin delineation during EMR and complement CADe algorithms by helping to exclude false-positive findings.

Hussein and colleagues implemented a CADx model based on I-Scan that, through leave-one-out cross-validation on a dataset comprising both still images and full video sequences of 57 patients, demonstrated high diagnostic accuracy with processing speeds compatible with real-time application (38). A comparable performance was obtained by Jukema and colleagues using NBI (39). These studies rely on high-quality images in an *ex vivo* setting and do not include cases of LGD, primarily due to the lack of a robust histopathological ground truth, which limits the development and validation of AI models targeting this early and clinically ambiguous stage.

Beyond lesion characterization, AI may also assist with determining the optimal therapeutic approach by predicting key pathological features such as the depth of invasion, presence of lymph vascular infiltration and lesion size. Identifying submucosal invasion is crucial as endoscopic submucosal dissection may be a valid alternative to surgery in selected T1b lesions, particularly when histopathologic criteria such as submucosal invasion < 500 μm, well or moderately differentiated histology and absence of lymph vascular invasion are met.

Currently, the distinction between T1a and T1b lesions relies primarily on optical evaluation, as endoscopic ultrasound (EUS) has limited accuracy for T-staging in this setting. When predicting T1b versus T1a stage based on expert assessment of endoscopic images, the overall sensitivity has been reported as low as 43.8%. This highlights a considerable risk of under-staging T1b disease using visual assessment alone (40).

In this context, AI-based may provide significant clinical value. A notable example is the DL model developed by Ebigbo and colleagues, which was trained on retrospectively collected Olympus WLE images to differentiate between T1a and T1b lesions. The model achieved a sensitivity of 77%, a specificity of 64% and an accuracy of 71%—comparable to that of an expert endoscopist with no statistically significant differences. This model demonstrates a high potential even because still images do not fully reflect real-world clinical assessment, where experienced endoscopists integrate dynamic features—such as esophageal wall movement, tissue pliability, and lesion behavior during insufflation and deflation—into their decision-making (41).

A summary of relevant recent studies on AI in the evaluation of BE associated dysplasia-neoplasia is represented in Table 1.

**TABLE 1:** Relevant recent studies on AI in the evaluation of BE associated dysplasia-neoplasia.

References	Study design	Study purpose	Training set	Test set	Modality	Endoscopic platforms	Diagnostic performance (Acc/Sen/Spe)	Updates
De Groof et al. (25)	Pilot study	Detection of BE-related neoplasia in live procedures	CAD trained on 494,364 GI + 1,544 BE images; tested on 2 external sets (∼160 patients)	Live WLE images from 20 patients (10 neoplastic, 10 non-dysplastic)	HD-WLE	Fujifilm	90%/91%/89%	First application of still-image CADe during live endoscopy
Ebigbo et al. (28)	Prospective study	Detection of BE-related neoplasia in live procedures	129 Endoscopic images	62 Endoscopic images assessed during live endoscopy	HD-WLE	Olympus	90%/84%/100%	Faster CADe But pausing of live video is required
Hashimoto et al. (37)	Pilot study	Detection of BE-related neoplasia in live procedures	9,016 Endoscopic images	458 Endoscopic images assessed during live endoscopy	WLE and NBI	Olympus	95%/96%/94%	Faster CADe No pausing required. Larger dataset
Abdelrahim et al. (29)	Multicentre trial	Detection of BE-related neoplasia	1,090,171 (Images and videos)	75 Videos 471 images	HD-WLE	Fujifilm, Olympus, Pentax	92%/94%/91%	Multicentre (3 centers), videos-based CADe, Different platforms
Fockens et al. (33)	Large multicenter trial	Detection of BE-related neoplasia	14,046 images across 15 centers	439 Videos 809 images	HD-WLE	Olympus	–/95%/- (images) –/97%/- (videos)	Multicentre (15 centers) videos-based CADe

### 2.2 Esophageal squamous dysplasia and carcinoma

Esophageal Squamous cell carcinoma (ESCC) is frequently diagnosed at an advanced stage, mainly because superficial lesions are typically asymptomatic and associated with only subtle mucosal changes which cannot be readily identified by WLE.

Narrow-band imaging (NBI), a non-invasive optical technique that enhances the superficial structure and microvascular pattern, is currently the standard endoscopic technique for detecting superficial ESCC (42). Despite its reported utility, diagnostic sensitivity remains low among less experienced endoscopists. To address this variability, in Eastern countries, AI-based systems have been developed to standardize and enhance detection accuracy.

A meta-analysis (43) of 3 studies (44–46) evaluated a total of 176,841 images from 218 patients in the test sets reporting a sensitivity of 93%, a specificity of 89% and a negative predictive value of 97% for ESCC detection. However, the overall methodological quality of the included studies was considered low due to potential selection and operator bias in the training sets used. To address limitations, Waki et al. assessed an AI system using unedited endoscopic videos that simulated real-world scenarios in which superficial esophageal squamous neoplasia (ESCN) could be overlooked—for instance, maintaining a uniform withdrawal speed without focused attention on specific mucosal areas. In their study, the AI system achieved a sensitivity of 85.7%, outperforming the average sensitivity of 21 participating endoscopists (47). Similarly, Shiroma et al. (48) aimed to simulate more realistic clinical conditions, where endoscopists often have limited time to inspect the entire esophagus testing a DL model using high speed video footage. The system demonstrated good performance, accurately detecting all ESCCs in slow-motion videos and 85% of lesions in fast-motion videos. However, like the system developed by Waki, it failed to detect precancerous lesions, thereby limiting its clinical utility for early-stage esophageal squamous cell carcinoma (ESCC) screening. Moreover, both AI systems remain at the experimental stage, have not yet been integrated into endoscopic platforms and have not been evaluated in real-world clinical trials.

More recently, Yuan et al. (49) conducted a multicenter randomized controlled trial across 12 hospitals in China to evaluate the diagnostic utility of an AI system for detecting ESCC and precancerous lesions in a real world clinical setting. Although the primary endpoint -reduction in miss rate per lesion or per patient in the AI-first group- did not reach statistical significance, the results remain clinically promising. The lack of significance may be attributed to the high expertise level of participating endoscopists, with nearly 80% being senior or intermediate-level practitioners and over 80% of hospitals being tertiary centers. The potential benefit may be more pronounced in lower resource settings (50).

Similarly, another randomized controlled trial conducted in a high-incidence region of China evaluated the real-time performance of the ENDOANGEL- DL. The study demonstrated a two-fold increase in the detection rate of high risk esophageal lesions in the AI assisted group compared to the control group (1.8% vs. 0.9%), with statistical significance (*p* = 0.029) (51).

A concrete example of the clinical effectiveness of AI-assisted endoscopy is reported by Zhou and colleagues who reported the incidental real-time detection of a subtle, flat-type ESCC during clinical endoscopy using an AI-based multimodal system, which was then confirmed to be high grade squamous intraepithelial neoplasia (52).

Beyond detection, accurate characterization of lesion depth using narrow-band imaging (NBI), particularly with magnifying endoscopy (NBI-ME), is crucial for determining the most appropriate treatment strategy.

Shimamoto et al. (53) used NBI video images to distinguish between mucosal/submucosal invasion (SM1) and submucosal deep invasion (SM2-3). Their CNNs model achieved a sensitivity of 90.1% and specificity of 95.8%, outperforming 16 experienced endoscopists (sensitivity of 89.8% and specificity of 88.3%).

These findings highlight the growing potential of AI to not only detect superficial ESCC but also to assist clinicians in real-time decision-making.

### 2.3 Gastric cancer

According to the Correa hypothesis, intestinal-type gastric cancer develops through a multistep inflammatory process of chronic gastritis, atrophic gastritis, intestinal metaplasia, dysplasia and carcinoma. Among these stages, gastric atrophy and intestinal metaplasia are considered as precancerous conditions.

#### 2.3.1 Precancerous conditions: challenges and advances in detection

Conventional white light endoscopy demonstrates only moderate sensitivity and specificity in the detection of gastric atrophy (GA) and intestinal metaplasia (IM) (54). Its diagnostic reliability is further compromised by substantial interobserver variability, particularly in cases with subtle or patchy mucosal changes.

A recent multicenter retrospective study conducted in countries with low gastric cancer incidence reported a sensitivity for detecting atrophic gastritis and gastric intestinal metaplasia to be as low as 48.5 and 16.3%, respectively (55). To overcome these challenges, deep learning models have emerged as promising tools. Guimarães and colleagues developed a DL-based algorithm trained on real world endoscopic images of the proximal stomach (corpus and fundus), achieving a diagnostic accuracy of 93%, which significantly exceeded the performance of experienced endoscopists in a tertiary referral center in detecting atrophic gastritis (56). Due to the small sample size, these results should be interpreted with caution when making generalizations.

Xu and colleagues employed ENDOANGEL, a real time AI system based on a deep convolutional neural network, to detect GA and IM using image-enhanced endoscopy (IEE). It demonstrated high diagnostic accuracy for GA (90.1% internal, 86.4% external, and 87.8% in prospective video sets) and for IM (90.8% internal, 85.9% external, and 89.8% in prospective video sets). However, low-grade dysplasia was not included due to insufficient data (57).

Additionally, a recent single-center randomized trial demonstrated that AI assistance significantly improved the detection rates of IM (from 9.2 to 14.2%) and GA (from 17.3 to 22.8%) while reducing blind spot rates by 46% (58).

Such technologies may be particularly valuable in standardizing surveillance practices and enhancing diagnostic performance in regions with limited endoscopic expertise.

#### 2.3.2 AI assisted detection of gastric neoplasm: improving early diagnosis

Gastric cancer ranks among the five most common malignancies worldwide and represents a significant burden on healthcare systems. Early diagnosis is crucial to improve patient outcomes, while the overall 5-year survival rate for early GC exceeds 90%, it drops to below 50% for advanced stages due to the disease’s aggressive nature and rapid progression. Despite its widespread use, white light endoscopy demonstrates limited efficacy in identifying early gastric cancer, with a pooled sensitivity of 48% and a specificity of 67%, underscoring the need for more effective diagnostic strategies.

ENDOANGEL is an AI-assisted system design to support physicians during endoscopic procedures by offering real-time guidance. It has demonstrated superior performance to endoscopists in detecting early gastric cancers (EGC) and comparable accuracy in assessing the invasion depth, differentiation status and tumor margins (59–61).

Furthermore, a recent randomized controlled trial showed that ENDOANGEL significantly reduced gastric neoplasm miss rates in clinical practice, with a reported relative risk of 0.224. This effect was particularly evident for small and antral lesions and among less experienced endoscopists, suggesting a valuable role for AI in standardizing diagnostic quality and enhancing early cancer detection in routine clinical practice. Remarkably, these results were achieved using only WLE, underscoring the system’s effectiveness even without advanced imaging modalities. However, as the study was conducted at a single center, its generalizability remains to be determined (62–64).

In a separate multicenter study, Luo and colleagues proposed and validated the GRAIDS system for the diagnosis of upper gastrointestinal neoplasms using a dataset of 1,036,496 endoscopic images from 84,424 patients. External validation was conducted across five primary care hospitals. The performance of GRAIDS was compared against that of endoscopists with three levels of expertise: expert, competent and trainee. GRAIDS demonstrated high diagnostic accuracy (up to 97.7%) and achieved sensitivity comparable to that of expert endoscopists while outperforming those with lower levels of experience (65). The systems’ high performance across diverse clinical settings supports its potential for real world integration, especially in resource-limited environments.

Lesion invasion depth represents one of the most important prognostic factors in the management of gastric cancer, as it directly influences therapeutic decisions, including the eligibility for endoscopic resection. EUS has been regarded as a valuable tool for this purpose; however, its diagnostic performance is limited by the operator’s experience and training, additional procedural time and financial burden. Additionally, studies have shown that EUS does not consistently outperform conventional endoscopy in assessing invasion depth, having a reported accuracy of approximately 70% (66).

Given these limitation, recent efforts have explored the application of AI systems.

Zhu and colleagues developed a CNN-CAD model with an AUC of 0.94 (CI 0.90–0.97) indicating its high discriminatory ability. Furthermore, it showed a significant improvement in accuracy (by 17.3%) and specificity (by 32.3%) compared to human endoscopist (67). Similarly, Chen and colleagues developed and AI system using WLE which demonstrated a diagnostic accuracy of 86.18%, significantly outperforming conventional methods. This highlights the promising role of AI as a reliable and objective tool for early gastric cancer staging (68).

#### 2.3.3 AI in the endoscopic diagnosis of *Helicobacter pylori* infection

Given that *Helicobacter pylori* (*H. pylori*) infection is a well-established precursor of gastric cancer and a key driver of chronic gastritis and intestinal metaplasia, its accurate and timely detection remains essential for effective risk stratification and prevention strategies. AI, especially deep learning models trained on image-enhanced endoscopy techniques such as LCI and Blue Laser Imaging (BLI), has substantially improved the endoscopic assessment and detection of *H. pylori* infection. These AI systems leverage the enhanced mucosal visualization and color contrast provided by LCI and BLI, allowing for more accurate identification of subtle mucosal changes associated with *H. pylori* infection, post-eradication status, and premalignant conditions such as atrophic gastritis and intestinal metaplasia (69). Notably, Nakashima et al. demonstrated that the diagnostic accuracy of the LCI-CAD system was 84.2% for uninfected, 82.5% for currently infected, and 79.2% for post-eradication status. These results were superior to those obtained using white-light imaging (WLI) with CAD, and the LCI-CAD system achieved diagnostic accuracy comparable to experienced endoscopists when using LCI images (70).

These advances hold meaningful implications for gastric cancer prevention. More precise AI-assisted detection of *H. pylori* status and residual mucosal abnormalities enables better risk stratification and tailored surveillance, especially for individuals with advanced atrophy or intestinal metaplasia, which are optimally visualized with LCI and BLI (69). In addition, AI-supported assessment may help standardize diagnosis and expand access to high-quality risk evaluation in resource-limited settings, supporting earlier intervention and more personalized screening pathways.

## 3 AI in advanced endoscopic imaging for lower GI

### 3.1 Adenomas and serrated lesions

#### 3.1.1 AI-based real-time detection and classification of adenomas and serrated lesions

AI is revolutionizing GI endoscopy by enhancing lesion detection and classification, particularly in colorectal cancer (CRC) prevention. Among the key targets of AI-assisted endoscopy are adenomas and serrated lesions, two distinct precancerous polyps with different biological behaviors (71).

Adenomas, characterized by dysplastic epithelial proliferation, follow the traditional adenoma-carcinoma sequence, typically involving mutations in the APC gene and the Wnt signaling pathway (72). They are generally considered to have a higher risk of progression to CRC if left untreated (72). While, serrated lesions, on the other hand, represent a distinct pathway to colorectal carcinogenesis called the serrated neoplasia pathway. They include hyperplastic polyps, sessile serrated lesions (SSLs), and traditional serrated adenomas (73). SSLs, in particular, are recognized as important precursors of CRC, especially in the proximal colon, and they often lack the classic dysplasia seen in adenomas but can harbor molecular alterations such as BRAF mutations and CpG island methylator phenotype (73). SSLs are more challenging to detect due to their flat morphology, predilection for the proximal colon, yet their early recognition is crucial, as they account for a significant proportion of interval CRC (74).

Beyond detection, accurately differentiating adenomas from SSLs is essential, as current guidelines recommend distinct endoscopic management strategies (75). Considering sessile or flat lesion, SSLs require different endoscopic management depending on the presence of dysplasia. For SSLs without dysplasia, the preferred approach is piecemeal cold snare polypectomy, especially for lesions larger than 10–15 mm. For SSLs with dysplasia or adenomatous lesions, the management approach changes based on lesion size. For intermediate-sized lesions (10–19 mm), hot snare polypectomy is preferred, with submucosal injection to minimize the risk of deep thermal injury. When the lesions are larger (≥ 20 mm), EMR is recommended for complete resection. For lesions ≥ 25–30 mm, piecemeal EMR is performed along with thermal ablation of the resection margins to reduce recurrence, considering en-bloc resection of the dysplastic area for SSLs. It is also important to accurately assess the depth of submucosal invasive carcinoma (SMIC), as some lesions with superficial invasion may be amenable to advanced endoscopic treatments such as endoscopic submucosal dissection (ESD), whereas those with deep invasion often require surgical management (75).

AI-driven polyp detection and classification systems offer a promising solution by improving the accuracy of identification, reducing missed lesions, and guiding optimal treatment and surveillance strategies.

CADe systems have been widely studied for their ability to assist endoscopists in identifying polyps during colonoscopy. Several platforms have received regulatory approval or are commercially available (Table 2).

**TABLE 2:** Commercially available computer-assisted endoscopy tools.

Computer assisted system	Product	Manufacturer	Year of regulatory approval	Place of regulatory approval
CADx	EndoBRAIN	Cybernet System Corp./Olympus Corp.	2018	Japan
CADe	GI-Genius	Medtronic Corp.	2019 in Europe; 2021 in United States	Europe/United States
CADe	ENDO-AID	Olympus Corp.	2020	Europe
CADx/CADe	CAD EYE	Fujifilm Corp.	2020	Europe/Japan
CADe	DISCOVERY	Pentax Corp.	2020	Europe
CADe	EndoBRAIN-EYE	Cybernet System Corp./Olympus Corp.	2020	Japan
CADe	EndoAngel	Wuhan EndoAngel Medical Technology Company	2020	China
CADe	EndoScreener	WISION A.I.	2020	China
CADx	EndoBRAIN-PLUS	Cybernet System Corp./Olympus Corp.	2020	Japan
CADx	EndoBRAIN-UC	Cybernet System Corp./Olympus Corp.	2020	Japan
CADe	WISE VISION	NEC Corp.	2021	Europe/Japan
CADe	ME-APDS	Magentiq Eye	2021	Europe
CADe	CADDIE	Odin Vision	2021	Europe
CADx/CADe	OLYSENSE (CADDIE, CADU, SMART IBD)	Olympus Corporation (Odin Medical Ltd., an Olympus company)	2024	Europe/United States

In parallel, CADx tools are being developed to support the real-time optical characterization and histological prediction of colorectal lesions, a task especially relevant for implementing “resect and discard” or “diagnose and leave” strategies (76). AI models trained using deep learning, including CNNs and transformer-based architectures, have shown promising results in distinguishing neoplastic vs. non-neoplastic polyps, in defining the grade of dysplasia and the invasion of submucosa layers, and more recently, in differentiating adenomas from sessile serrated lesions, which is a known challenge in routine practice due to their subtle and often flat appearance.

For example, in a prospective pilot study involving 128 patients and 209 polyps detected via narrow-band imaging (NBI), an AI model was developed based on three key features: mean vessel length, vessel circumference, and brightness within detected vessels (77). The CADx system achieved a sensitivity of 90% and specificity of 70.2% in distinguishing neoplastic from non-neoplastic polyps, although endoscopists performed better, with up to 96.9% sensitivity and 85.7% specificity in cases with interobserver agreement. Despite a correct classification rate of 85.3%, the system was not fully automated and its performance in real-time clinical settings remains uncertain. Additionally, reliance on NBI limits generalizability to environments where this modality is unavailable.

Real-time application of CADx is essential for its clinical integration. Some models using real-time support vector machine-based outputs have shown encouraging results (78–82). For example, Chen et al. (83) demonstrated a CADx system using NBI that predicted the histology of 284 diminutive polyps, 1–5 mm (96 hyperplastic, 188 neoplastic) with 96.3% sensitivity, 78.1% specificity, and a negative predictive value (NPV) of 91.5%, surpassing the ASGE PIVI threshold (NPV ≥ 90%) for the “diagnose and leave” approach (84). Similarly, Byrne et al. reported strong outcomes using combined CADe and CADx systems (85). However, these findings require confirmation in larger, controlled trials to mitigate selection bias and better assess performance in routine practice.

Although some prospective studies have demonstrated favorable diagnostic performance for CADx systems (86, 87) variability remains. For instance, the CADx models evaluated by Kuiper et al. failed to reliably distinguish adenomatous from non-adenomatous polyps (88). Another prospective study by Rath et al. reported moderate diagnostic metrics (accuracy 84.7%, sensitivity 81.8%, specificity 85.2%, NPV 96.1%), suggesting suitability for implementing a “diagnose and leave” approach in rectosigmoid polyps (89). However, a low prevalence of neoplastic lesions may have inflated the NPV while underestimating the model’s overall accuracy and positive predictive value.

Regarding characterization of dysplasia, a study by Shimizu et al. developed a deep learning model utilizing NBI to classify colorectal tumors into four histologic categories—low-grade dysplasia, high-grade dysplasia, superficial submucosal invasion, and deep submucosal invasion—with image-level accuracies ranging from 96.4 to 99.2% (90).

More recently, AI models capable of differentiating SSLs from other polyp types have been proposed. Particularly, a study evaluated the POLAR CADx system for real-time optical diagnosis of diminutive colorectal polyps, including 41 SSLs (91). While POLAR showed comparable overall accuracy to endoscopists (79% vs. 83%), its sensitivity for identifying SSLs was significantly lower (17.1% vs. 58.6%).

Despite these advances, the classification of SSLs remains more challenging than that of adenomas, due in part to their underrepresentation in training datasets and subtle visual features. Therefore, current research is focusing on improving dataset diversity, incorporating multi-center image repositories, and refining model interpretability to support adoption in clinical workflows. Continued validation in prospective trials will be essential to confirm the clinical utility of CADx systems for both adenomas and serrated lesions and their impact on CRC prevention. Furthermore, multimodal approaches combining image features with clinical data (e.g., size, location, patient age) are being investigated to enhance model robustness (92).

#### 3.1.2 Impact of AI-assisted colonoscopy on adenoma detection rates

The detection of colorectal neoplasia using CADe systems has been extensively studied through numerous randomized controlled trials (RCTs) (93–96). A recent and comprehensive meta-analysis published in the Annals of Internal Medicine in 2024 by Soleymanjahi et al. provided robust evidence supporting the effectiveness of CADe in enhancing polyp detection during colonoscopy (97). This meta-analysis included 44 RCTs comparing standard colonoscopy with CADe-assisted procedures across patients undergoing screening, surveillance, or diagnostic evaluations. Multiple CADe platforms were assessed, including GI Genius (Medtronic), Fujifilm CAD EYE, Endo Screener (developed using the SegNet architecture), and systems based on the You Only Look Once (YOLO) neural network. All studies used high-definition colonoscopy equipment. Despite the diversity in CADe algorithms and system architectures, consistent improvements in adenoma detection were observed. Specifically, CADe increased adenoma detection rates (ADR) from 36.7 to 44.7% (RR 1.21; 95% CI, 1.15–1.28) and adenomas per colonoscopy (APC) from 0.78 to 0.98 (incidence rate difference 0.22; 95% CI, 0.16–0.28). Additionally, in tandem colonoscopy trials, CADe significantly reduced adenoma miss rates (AMR), from 35.3 to 16.1% (RR 0.47; 95% CI, 0.36–0.60). When examining individual systems, those based on YOLO showed a particularly marked benefit, with ADRs increasing from 22 to 29% (RR 1.36; 95% CI, 1.14–1.62). GI Genius raised ADRs from 50 to 55% (RR 1.16; 95% CI, 1.00–1.34), Fujifilm CAD EYE from 43 to 53% (RR 1.21; 95% CI, 1.10–1.34), and EndoScreener from 26 to 31% (RR 1.22; 95% CI, 1.11–1.35) (97).

These results build on earlier findings from a 2023 meta-analysis by Hassan et al., which also demonstrated a significant improvement in ADR—from 35.9 to 44.0% (RR 1.24; 95% CI, 1.16–1.33)—and a 55% reduction in AMR (RR 0.45; 95% CI, 0.35–0.58) with CADe use (16).

Further supporting evidence comes from a meta-analysis by Maida et al., which specifically evaluated CADe performance in comparison to standard white-light colonoscopy (WLC). This analysis of six tandem RCTs involving 1,718 patients revealed that CADe reduced AMRs by 54% and polyp miss rates (PMR) by 56%. Notably, the analysis showed low heterogeneity (18%) and consistent results across studies, reinforcing the robustness of the findings. A sensitivity analysis restricted to screening and surveillance populations confirmed CADe’s benefit in reducing missed adenomas and polyps, strengthening its role in improving the accuracy of colorectal cancer screening (98).

However, the Soleymanjahi et al. meta-analysis also identified considerable heterogeneity in CADe’s effect on ADR (97). Importantly, this heterogeneity was mainly due to variations in the magnitude of benefit rather than conflicting outcomes—40 out of 44 studies favored CADe-assisted colonoscopy. Two key factors may account for this variability. First, a recent meta-regression of 23 RCTs suggested that much of the heterogeneity stemmed from differences in colonoscopy quality, particularly baseline ADRs in control groups and withdrawal times (99). Second, the clinical setting may influence CADe’s performance. For example, trials involving FIT-positive populations or patients with Lynch syndrome—where advanced adenomas were the primary focus—did not show significant ADR improvements, highlighting limitations in these specific high-risk subgroups (95, 100).

AI-powered systems for polyp detection and classification represent a promising advancement by enhancing the accuracy of lesion identification and characterization, minimizing the risk of missed lesions, and supporting more effective treatment and surveillance decision-making. This is particularly important given the growing body of research identifying serrated lesion as a major contributor to CRC, potentially accounting for up to 35% of all cases, and their frequent occurrence as interval cancers in the proximal colon (101).

Recent meta-analyses suggest that CADe systems may help improve the detection of SSLs. For instance, Soleymanjahi et al. reported a 21% relative increase in SSL detection rates with CADe compared to standard colonoscopy (RR: 1.21, 95% CI: 1.04–1.42). However, the absolute gain remains modest, with an incidence rate difference (IRD) of only 0.02 SSLs per colonoscopy (95% CI: -0.01 to 0.04) (97). This modest improvement underscores the current limitations of CADe systems in reliably detecting SSLs, particularly in the proximal colon, where these lesions are more frequently overlooked. One possible explanation is the underrepresentation of SSLs in the training datasets used to develop CADe algorithms, which may hinder their ability to accurately identify the distinct characteristics of these lesions (102). To overcome this challenge, future efforts should prioritize the inclusion of a greater number of flat, serrated, and advanced adenomas in training datasets. This will be essential for future research aimed at evaluating the true clinical impact of CADe on detecting high-risk lesions and ultimately improving colorectal cancer prevention.

A summary of the studies involving computer-aided diagnosis for colonoscopy including studies with combined detection and diagnosis systems are represented in Table 3.

**TABLE 3:** Summary of the studies involving computer-aided diagnosis for colonoscopy including studies with combined detection and diagnosis systems.

Author	Study design	Study aim	System	Modality	Number of patients/ colonoscopies used for training/test datasets (total)	Number of colonoscopy/polyp images/videos used in training/test datasets	Diagnostic properties
Tischendorf et al. (77)	Prospective pilot	Distinguishing adenomas from non-adenomas	CADx based on SVMs	WLE and NBI	NA/128; Colonoscopy videos	NA/209 polyps containing 160 neoplastic and 49 non-neoplastic polyps in the test dataset	Computer-based approach achieved a sensitivity ≈ 90% and a specificity ≈ 70%. Overall correct classification rate = 85.3%.
Chen et al. (83)	Retrospective comparative study	analyze narrow-band images of diminutive colorectal polyps	DNN-CADx	NBI	NA	2,157 NBI images (1,476 of neoplastic polyps and 681 of hyperplastic polyps)	DNN-CAD identified neoplastic or hyperplastic polyps with 96.3% sensitivity, 78.1% specificity, a PPV of 89.6%, and a NPV of 91.5%
Byrne et al. (85)	Retrospective	Distinguishing diminutive (≤ 5 mm) neoplastic from non-neoplastic lesions	CADx based on DCNN	NBI	NA	Training dataset: 60,089 frames from 223 polyp videos (29% NICE type 1, 53% NICE type 2 and 18% of normal mucosa with no polyp)/validation dataset: 40 videos (NICE type 1, NICE type 2 and two videos of normal mucosa)/test dataset: 125 consecutively identified diminutive polyps, comprising 51 hyperplastic polyps and 74 adenomas	Accuracy = 94%, sensitivity = 98%, specificity = 83%, NPV = 97%, PPV = 90%
Aihara et al. (86)	Prospective	Distinguishing neoplastic from non-neoplastic lesion	CADx based on numerical color analysis of autofluorescence endoscopy as an Adobe AIRapplication	Autofluorescence endoscopy (AFE)	NA/32 patients in the test dataset	NA/102 lesions containing 75 neoplastic lesions in the test dataset	Sensitivity = 94.2%, specificity = 88.8%, PPV = 95.6%, NPV = 85.2%
Mori et al. (87)	Prospective	Distinguishing diminutive (≤ 5 mm) neoplastic from non-neoplastic lesions	CADx based on SVMs used with NBI and endocytoscope	WLE, NBI, endocytoscopy with methylene blue staining	NA/791 patients in the test dataset	61,925/466 polyps from 325 patients in the test dataset	CADx-NBI: Sensitivity = 92.7%, specificity = 89.8%, PPV = 93.7%, NPV = 88.3% CADx-endocytoscope: Sensitivity = 91.3%, specificity = 88.7%, PPV = 92.9%, NPV = 86.3%
Kuiper et al. (88)	Retrospective	Distinguishing small (≤ 9 mm) neoplastic from non-neoplastic lesion	CADx (WavSTAT) based on CNN	Laser-induced autofluorescence spectroscopy	NA/87 patients in the test dataset	NA/207 small lesions in the test dataset	Accuracy = 74.4%, sensitivity = 85.3%, specificity = 58.8%, PPV = 74.8%, NP = 73.5%; accuracy of on-site recommended surveillance interval = 73.7%
Rath et al. (89)	Prospective	Accuracy of LIFS-based optical biopsy system in predicting the histology of diminutive colorectal polyps	WavSTAT4 optical biopsy system	Laser-induced fluorescence spectroscopy (LIFS)	NA/27 patients in the test dataset	NA/37 diminutive colorectal polyps in the test dataset	Accuracy = 84.7% with sensitivity = 81.8%, specificity = 85.2%, NPV = 96.1%
Shimizu et al. (90)	Retrospective	Develop and evaluate a deep learning model capable of classifying colorectal neoplastic lesions into four histologic categories—LGD, HGD, SMs and SMd—using NBI images	A patch-based classification model employing a residual CNN	NBI	NA	NA/1,390 NBI images (294 images of LGD, 840 images of HGD, 138 images of SMs, 118 images of SMd)	Accuracy was 0.876, 0.957, 0.907 and 0.929 in LGD, HGD, SMs and SMd, respectively
Houwen et al. (91)	Prospective	Compare the accuracy of the optical diagnosis of diminutive colorectal polyps, including SSLs, between CADx system and endoscopists during real-time colonoscopy.	POLyp Artificial Recognition (POLAR) system	WLE, NBI, i-SCAN, and LCI	NA	2,637 NBI images from 1,339 polyps (73% adenomas, 10% SSLs, 17% hyperplastic polyps)/423 diminutive polyps	POLAR distinguished neoplastic from non-neoplastic lesions with 79% accuracy, 89% sensitivity, and 38% specificity. The endoscopists achieved 83% accuracy, 92% sensitivity, and 44% specificity. The proportion of polyps in which POLAR was able to provide an optical diagnosis was 98%

SVM, Support vector machine; DNN, deep neural network; NBI, narrow-band imaging; DCNN, Deep learning convolutional neural network; PPV, Positive predictive value; NPV, Negative predictive value; CNN, Convolutional neural network; LIFS, laser-induced fluorescence spectroscopy; LGD, low grade dysplasia; HGD, high grade dysplasia; SMs, superficially invasive submucosal carcinoma; SMd, deeply invasive submucosal carcinoma.

### 3.2 AI in IBD

The application of AI in inflammatory bowel diseases (IBD) has gained increasing attention in recent years, offering new opportunities to enhance diagnostic accuracy, predict disease progression, and personalize treatment strategies (103). By leveraging large datasets from endoscopic imaging, histology, clinical records, and omics technologies, AI models are being developed to support clinicians in distinguishing disease phenotypes, assessing mucosal healing, and forecasting therapeutic response. These innovations hold the potential to improve patient outcomes and streamline decision-making in the complex management of IBD.

#### 3.2.1 AI in ulcerative colitis

Endoscopic remission is considered a long-term treatment goal in ulcerative colitis (UC) given its association with favorable long-term clinical outcomes (104). Moreover, histological remission has become increasingly important (105). White light endoscopy (WLE) is the mainstay for assessing UC disease activity and treatment efficacy. The most common endoscopic scoring systems to determine UC grade of inflammation and remission are the Mayo Endoscopic score (MES) and Ulcerative Colitis Endoscopic Index Severity (UCEIS) (106). Based on WLE findings, these scores are routinely adopted in clinical practice and central trial readouts. However, these scores are unreliable for accurately defining mucosal healing or remission. Furthermore, the WLE assessment is not failproof and discrepancies persist between endoscopic activity assessment and histology (107), mainly due to the patchiness of inflammation and random biopsy sampling instead of targeted biopsies. The major limitation of endoscopic scores is their high intra- and inter-rater variability among endoscopists due to unavoidable subjectivity. AI has demonstrated remarkable potential in assisting and expediting image analysis, potentially addressing many challenges in disease assessment. AI-enabled endoscopy and histology allow rapid, objective, and accurate evaluation of disease activity and prediction of UC outcomes. An AI model could greatly and “real-time” enhance the accuracy and depth of disease evaluation, offering a more nuanced and rapid assessment of histological healing in UC.

Considering that, in 2019, Ozawa et al. introduced a CNN-CAD system, which, using deep learning, accurately classified 73, 70, and 63% of images corresponding to MES 0, MES 1, and MES 2–3, respectively, when benchmarked against expert evaluation (108). Concurrently, Stidham et al. developed a CNN capable of a four-level classification of UC severity (MES 0–3), demonstrating robust performance in distinguishing among these grades (109). Takenaka et al. also proposed a deep learning model that could identify endoscopic remission with 90% accuracy and predict histological remission with 92% sensitivity and 94% specificity from endoscopic images (110). In a subsequent multicentre prospective study, an updated video-based version of their algorithm achieved even higher diagnostic accuracy, with 98% sensitivity and 95% specificity for histological remission (111). However, these initial studies primarily relied on still images, raising concerns about potential selection bias. To overcome this, Yao et al. designed an AI system capable of autonomously analyzing unedited endoscopy videos and generating summary scores for entire procedures, automatically excluding suboptimal frames to increase diagnostic reliability (112). Similarly, Takenaka et al. adapted their model for video input, confirming its correlation with expert assessments of endoscopic remission (110). Recently, three systematic reviews and meta-analyses confirmed the high accuracy of AI in detecting endoscopic remission using both still images and videos, based on MES and UCEIS criteria, while noting moderate to high heterogeneity and a potential risk of bias, highlighting the need for higher-quality validation studies (113–115).

Beyond remission detection, AI also enhances assessment of disease extent and patchiness—key factors in tailoring treatment strategies. Notably, Takabayashi et al. (116) and Fan et al. (117) proposed new scoring systems that incorporate spatial distribution of inflammation for a more comprehensive disease activity profile. Stidham et al. introduced the Cumulative Disease Score, leveraging computer vision on endoscopy videos from the UNIFI trial (assessing ustekinumab in UC) to quantify mucosal damage more precisely than conventional MES scoring, improving evaluation of treatment efficacy (118). Furthermore, AI-driven approaches can streamline disease scoring in clinical trials, as shown by Gottlieb et al., who trained a CAD system using data from a phase II mirikizumab trial (119). Their model demonstrated high accuracy in scoring endoscopic activity and has the potential to standardize and accelerate central reading processes. More recently was developed an “AI switching model” which can identify the acquisition modality of each frame (WLE, iScan, and NBI) and convert the image to another modality, obtaining a wide range of simultaneous multiple endoscopic modalities during a single procedure (120). By integrating multimodal images, the model refines inflammation assessment and outcome prediction, supporting more precise disease stratification and treatment decisions. This multimodal AI approach also mitigates interobserver variability and subjectivity, which are persistent challenges in conventional endoscopic scoring, and facilitates real-time, objective evaluation of mucosal healing and disease activity.

The emergence of advanced endoscopic technologies, which allow for more precise and in-depth evaluation of the intestinal mucosa, has led to the integration of AI to enhance the assessment of histological healing. Iacucci et al. introduced an innovative AI-driven system that calculated the Paddington International virtual Chromoendoscopy ScOre (PICaSSO) score using iSCAN endoscopic videos, achieving accuracies of 83, 81, and 83% in identifying Robarts Histopathology Index (RHI) ≤ 3, Nancy Histological Index (NHI) ≤ 1, and PICaSSO Histological Remission Index = 0, respectively (121). Moreover, the AI-PICaSSO model demonstrated a robust correlation between AI-derived disease activity scores and adverse clinical outcomes. Furthermore, a recent systematic review and meta-analysis confirmed these findings, demonstrating that AI holds substantial potential for assessing histological remission in ulcerative colitis, with performance comparable to that of expert pathologists (122). AI-integrated advanced endoscopic techniques enable a more objective and detailed evaluation of mucosal healing in IBD, facilitating the assessment of histological remission and the prediction of clinical outcomes. This innovation holds the potential to transform precision medicine in the management of IBD. Ultimately, enhanced precision in defining inflammation distribution could refine colorectal cancer surveillance strategies in IBD, supporting earlier detection and improved clinical outcomes.

#### 3.2.2 AI in Crohn’s disease

In Crohn’s disease (CD), artificial intelligence has primarily been applied to capsule endoscopy (CE), a non-invasive modality essential for visualizing the small intestine. CE plays a pivotal role in assessing disease activity, detecting mucosal inflammation, and identifying complications such as strictures and fistulas (123). However, interpretation of CE imagery is often laborious and influenced by the clinician’s expertise. Recent advancements have integrated AI into CE analysis, enabling automated identification of IBD-related mucosal abnormalities, including ulcerations, erosions, and strictures. Klang et al. developed an AI algorithm that detected ulcers and strictures with AUCs of 0.94 and 0.99, respectively (124). Additionally, Majtner et al. demonstrated that their AI model could classify lesions into four categories—normal mucosa, aphthous ulcers, ulcers, and large ulcers/fissures—with substantial concordance with expert gastroenterologists (κ = 0.72) (125). These AI-driven systems enhance early diagnosis and facilitate longitudinal disease monitoring. Notably, AI-enhanced CE also shows prognostic utility: Kellerman et al. reported an algorithm capable of predicting the need for biologic therapy within 6 months of a new CD diagnosis, with an accuracy of 81% and an AUC of 0.86, outperforming clinicians and fecal calprotectin measurements (126). Furthermore, Brodersen et al. conducted a multicentre study employing the AXARO platform for pan-enteric CE analysis in patients with suspected CD, achieving 92–96% sensitivity and 90–93% specificity for CD detection, and 97% sensitivity with 90–91% specificity for IBD overall (127). AXARO also significantly reduced review time to a median of 3.2 min. Collectively, AI-assisted endoscopy offers an objective and efficient means of evaluating mucosal healing in both UC and CD, with promising applications in disease monitoring, relapse risk stratification, and therapy response prediction—supporting its potential integration into clinical practice and trials.

#### 3.2.3 AI in endoscopy for IBD surveillance and assessment of dysplasia

Patients with IBD have elevated risk of developing CRC (128), with incidence increasing proportionally with disease duration reaching approximately 1, 4, and 14% at 10-, 20-, and 30-years post-diagnosis, respectively. Detecting and grading IBD-associated dysplasia remains challenging owing to features such as chronic inflammation, flat lesion morphology, and poorly demarcated borders that blend with surrounding mucosa (128).

Surveillance colonoscopy in IBD patients is recommended, especially in those with long-standing UC or colonic CD. According to current international guidelines (ECCO, SCENIC), surveillance should begin 8 years after diagnosis in patients with extensive colitis and be repeated every 1–5 years based on individual risk factors (e.g., disease extent, severity of inflammation, family history, primary sclerosing cholangitis, previous dysplasia). High-definition endoscopy with chromoendoscopy [dye-based (DCE) or virtual (VCE)] is the preferred technique to enhance dysplasia detection. Targeted biopsies are favored over random sampling unless chromoendoscopy is not feasible (129, 130).

With the recent incorporation of AI into endoscopic practice, its potential for enhancing dysplasia detection in IBD is gaining attention. AI may improve early identification of IBD-related dysplasia, help identify patients requiring surveillance colonoscopy and support the development of tailored surveillance strategies. However, current evidence is limited to case reports and small-scale studies.

In two case reports, EndoBRAIN and EndoBRAIN-EYE CAD systems—originally validated for colorectal polyp detection—were applied during endocytoscopy and HD-NBI in two longstanding pan-colitis ulcerative colitis patients. Both systems successfully identified a neoplastic and a low-grade dysplastic flat lesion (131, 132).

Guerrero Vinsard et al. retrained a convolutional AI model for IBD-specific lesion detection and tested it on HD-WLE images of histologically confirmed dysplastic lesions amidst mild-to-moderate inflammation (133). The system demonstrated strong performance on HD-WLE images with 96.8% diagnostic accuracy and an AUC of 0.85, outperforming DCE-based evaluations (77.8% accuracy, AUC 0.65). Notably, the system showed greater sensitivity in detecting lesions ≤ 10 mm (93% for ≤ 5 mm, 91% for 6–10 mm, 85% for ≥ 10 mm), recognizing that larger IBD lesions are often pseudopolyps with complex features. Its sensitivity was higher for Paris classification types Ip, Is, and IIa, whereas IIb and mixed-morphology lesions were more frequently missed. Additionally, while it could detect serrated lesions, the true-positive rate (85.7%) was slightly lower than that for other lesion types (> 90%). Missed lesion rates also correlated with inflammation severity (7.3% for MES 0, 1.5% for MES 1, 8.7% for MES 2–3).

Another model using a RetinaNet architecture (ResNet-101 backbone) trained on 478 images from 30 IBD patients distinguished between “neoplastic” and “non-neoplastic” lesions with sensitivities of 93.5 and 87.5%, and a specificity of 80.6% for detection and classification, respectively (134). Furthermore, a CNN-based AI system (EfficientNet-B3) enabled binary classification of neoplasia in IBD into “adenocarcinoma/HGD” and “LGD/sporadic adenoma/normal mucosa.” This model outperformed both expert and non-expert endoscopists, achieving 79% accuracy (95% CI: 72.5–84.6), compared to 77.8% (95% CI: 74.7–80.8) and 75.8% (95% CI: 72–79.3), respectively (135). However, despite using p53 and Ki-67 immunostaining for lesion classification, the lack of molecular profiling limits the ability to exclude sporadic neoplasia. Such AI-based platforms may support less experienced endoscopists in recognizing colitis-associated dysplasia or CRC, potentially minimizing unnecessary biopsies.

### 3.3 AI in capsule endoscopy

AI, particularly deep learning–based systems, has rapidly advanced the field of CE by overcoming key limitations such as labor-intensive video interpretation and significant interobserver variability (136). Recent multicenter validation studies and meta-analyses show that AI-assisted CE achieves excellent performance in the detection of obscure gastrointestinal bleeding, including angioectasias, active bleeding, and subtle red spots, with results that often match or even surpass expert readers. Overall, AI-assisted CE reached a pooled sensitivity of 92.4% (95% CI: 86.5–98.7) for small-bowel lesion detection, compared with 86.0% (95% CI: 78.6–93.4) for conventional reading. While specificity was lower (53.7% vs. 99.8%), AI maintained strong predictive performance, with a PPV of 89.3% (95% CI: 75.5–99.9) and an NPV of 94.3% (95% CI: 93.9–94.6) (137).

Machine learning methodologies in video capsule endoscopy–based bleeding analysis have evolved substantially over the years, shifting from traditional color and texture–based algorithms to deep learning approaches. Convolutional neural networks (CNNs) and transfer learning now achieve sensitivity and specificity rates consistently above 95% for bleeding detection, with some models reaching 99% accuracy (138). These systems markedly reduce manual review time and interobserver variability and are increasingly robust across diverse datasets and imaging conditions. Real-time mobile applications leveraging ensembles of deep learning models, such as Faster R-CNN combined with LinkNet, allow frame-level classification and segmentation of bleeding versus non-bleeding frames with accuracies often exceeding 97%, enabling rapid, reliable clinical review and precise localization of bleeding regions (139).

Beyond bleeding detection, AI models also provide automated, reproducible scoring of bowel cleanliness using multi-label datasets, achieving accuracy rates up to 95% and closely matching expert assessment. This standardization supports objective quality control in CE interpretation, further enhancing efficiency and diagnostic reliability (140).

AI systems also demonstrate high accuracy in identifying ulcers, erosions, strictures, mucosal breaks, and small-bowel tumors, thereby improving diagnostic yield across a wide spectrum of small-bowel diseases. Notably, AI-assisted reading detected 96.1% of pleomorphic small-bowel lesions, compared with 76.3% using standard interpretation, with sensitivity rates of 97.5% versus 78.2%, respectively (141).

AI integration substantially reduces CE reading time by pre-screening videos, prioritizing abnormal frames, and generating automated lesion maps, thereby streamlining clinician review without compromising diagnostic concordance (142). In practical terms, AI-assisted reading can shorten interpretation time from nearly an hour to under 10 min per case while maintaining, and in some instances exceeding, standard lesion detection rates. For example, AI models developed for suspected small-bowel bleeding have achieved accuracies of 99.4% for erosions/ulcers, 99.8% for angiodysplasia, and 99.9% for active bleeding, with corresponding reductions in reading time from 53.9 to 8.7 min per case (143). Furthermore, AI algorithms can automatically filter poorly visualized or low-quality frames, enhancing efficiency and supporting consistent diagnostic performance (142).

Despite these advances, current AI systems are not yet fully autonomous; a proportion of lesions may still be missed, necessitating human oversight. Standardization across devices and further prospective clinical validation are required for routine clinical integration. Nonetheless, AI in CE is one of the fastest-growing areas in gastrointestinal imaging, with strong evidence supporting its transformative potential for improving efficiency, accuracy, and reducing interobserver variability in small bowel evaluation.

## 4 Discussion

**FIGURE 1:**
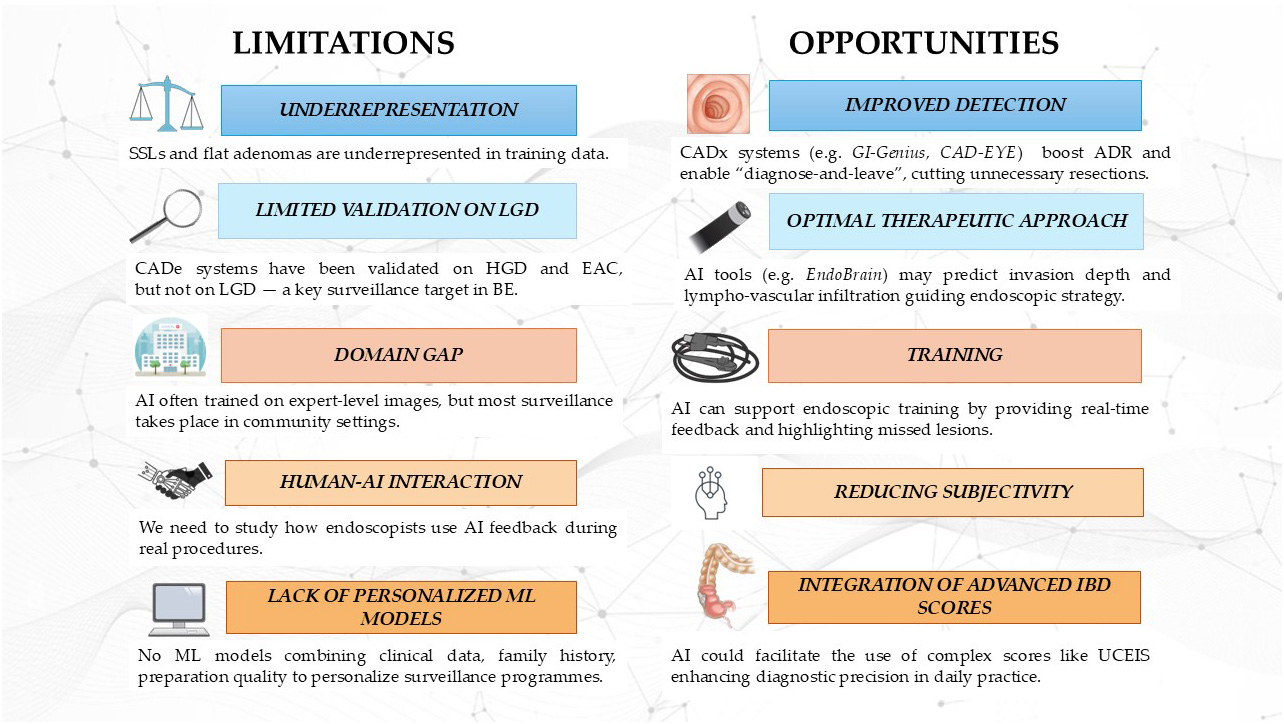
Limitations and opportunities of AI in GI endoscopy. SSL, Sessile Serrated Lesion; LGD, Low-Grade Dysplasia; HGD, High-Grade Dysplasia; EAC, Esophageal Adenocarcinoma; CADe, Computer-Aided Detection; CADx, Computer-Aided Diagnosis; ADR, Adenoma Detection Rate; IBD, Inflammatory Bowel Disease; UCEIS, Ulcerative Colitis Endoscopic Index of Severity.

In the past two decades, advances in endoscopic imaging have significantly enhanced mucosal visualization, enabling more accurate real-time lesion characterization and reducing the need for histological confirmation in selected cases (144). Yet, diagnostic accuracy continues to be hampered by interobserver variability and dependence on operator expertise, factors that limit reproducibility and generalizability in everyday practice (Figure 1).

### 4.1 AI in upper and lower GI

In the upper GI tract, AI shows promise in the surveillance of BE, particularly by assisting in the detection of early neoplasia and informing treatment decisions such as selecting between EMR and ESD. However, most current tools are primarily focused on identifying HGD and early adenocarcinoma, with limited performance in detecting LGD. This represents a significant limitation in the context of surveillance, where accurate identification of LGD could substantially influence follow-up intervals and clinical management. The lack of a consistent histopathological gold standard, due to high interobserver variability and frequent diagnostic reclassification, further complicates the use of LGD as a reliable target for AI training and validation. As a result, the development of CAD systems aimed at improving early neoplasia detection in BE remains hindered, limiting their potential to enhance surveillance strategies. Further compounding these challenges is the “domain gap” between the high-quality, curated datasets used to train AI models and the variable image quality encountered in real-life endoscopic practice. This discrepancy significantly limits the generalizability of AI systems. Evidence suggests that exposure to lower-quality images can markedly impair AI performance, with miss rates for neoplasia in early BE lesions reported to reach up to 53% (34). A similar challenge affects the development of AI for ESCC, which remains largely preclinical due to the scarcity of large, annotated datasets (*small data* problem) especially in low-incidence regions.

In the upper-GI domain, the role of EUS has recently gained renewed attention. AI is emerging as a promising adjunct in EUS for the evaluation of the esophagus and stomach, particularly for esophageal early cancer and gastrointestinal stromal tumors enhancing early cancer detection and lesion characterization (145, 146). However robust clinical validation and ethical oversight are necessary before routine clinical implementation.

In the lower GI tract, AI with CADe and CADx systems, has emerged as a powerful tool to support clinical decision-making and standardize lesion assessment. CADe systems have consistently shown benefits in improving lesion recognition and increasing ADR, a key quality indicator for colorectal cancer prevention. However, their true impact on CRC prevention remains uncertain. Many detected lesions are diminutive and carry limited oncologic relevance, whereas flat, serrated, or infiltrative lesions, particularly those located in the proximal colon, tend to be under detected, likely due to their underrepresentation in training datasets. These limitations are compounded by concerns over operator performance. Evidence suggests that routine use of CADe can lead to “deskilling,” with decreased vigilance and lower detection rates during unaided procedures (147, 148). This highlights the need for structured training, continuous quality monitoring and algorithm refinement including the development of larger, more diverse, and representative datasets to improve AI generalizability and performance. CADx systems, on the other hand, have shown high accuracy in differentiating neoplastic from non-neoplastic lesions across the GI tract. Some models report concordance rates above 90% with expert histopathological interpretation (83), supporting innovative approaches such as “resect-and-discard” and “diagnose-and-leave” in the management of diminutive polyps. In these scenarios, AI can enhance diagnostic precision while promoting cost-effective care and avoiding unnecessary resections (76).

Regarding IBD, AI in enhances diagnostic accuracy, enables real-time assessment of mucosal and histological healing, and supports prediction of treatment response, reducing interobserver variability (103). In UC, AI improves detection of endoscopic and histologic remission, while in CD it streamlines capsule endoscopy analysis and lesion monitoring. AI also shows potential in dysplasia surveillance, aiding early detection and risk stratification. However, limitations include reliance on high-quality data, potential bias from training datasets, and the need for further validation in large, multicenter studies.

### 4.2 The serrated lesion challenge

Serrated lesions account for approximately 35% of CCR and are a major contributor to interval cancers due to their challenging detection and characterization during colonoscopy (149). AI-assisted detection has shown only a modest 21% relative improvement over standard practice in reducing miss rates for sessile serrated lesions, as demonstrated in meta-analyses and multicenter randomized controlled trials (97). Despite this improvement, AI performance for serrated lesions remains suboptimal compared to its efficacy for conventional adenomas.

AI underperformance for serrated lesions is attributed to several factors: underrepresentation in training datasets, flat morphology, subtle color and textural differences, and variable presentation. These features make serrated lesions difficult for both human endoscopists and AI algorithms to reliably detect, especially in the proximal colon and in the presence of suboptimal bowel preparation (149, 150). The literature emphasizes the need for larger, more diverse datasets, targeted training on serrated lesion features, and prospective multicenter validation studies to enhance AI performance and reduce interval cancer rates associated with missed serrated lesions (151).

In summary, serrated lesions represent a significant challenge for AI-assisted colonoscopy, and current evidence supports the need for improved data diversity, algorithmic refinement, and rigorous prospective validation to address this gap and improve patient outcomes.

### 4.3 Human-AI collaboration

Endoscopists’ decisions are shaped not only by AI output, but also by cognitive factors such as automation bias, fatigue, time pressure, and even aversion to new technology (152). Although AI systems in endoscopy demonstrate high standalone accuracy, endoscopist performance often falls short due to automation bias and variable trust in AI outputs. Automation bias manifests as over-reliance, where clinicians may uncritically accept AI recommendations, and under-reliance, where valid AI alerts are dismissed, both of which can compromise diagnostic accuracy and patient safety (153, 154). Deskilling is a particular concern: recent multicenter evidence shows that routine AI-assisted procedures can reduce vigilance and manual detection skills, even among expert endoscopists (155, 156). Specifically, continuous AI exposure was associated with a decrease in ADR from 28.4 to 22.4%, a 6% absolute reduction during subsequent non-AI-assisted procedures, despite patient and procedural factors that would typically favor higher detection rates. To optimize human–AI interaction and mitigate these risks, the medical literature supports several strategies. Structured training programs that educate clinicians on AI capabilities, limitations, and error profiles are essential to reduce automation bias and foster appropriate trust (153, 154). Alert and interface design should prioritize actionable findings and minimize cognitive overload, while integration of AI into standardized workflows can help maintain operator engagement and oversight (157). Continuous performance monitoring is recommended to identify and address potential skill erosion, ensuring that AI augments rather than replaces clinical judgment (158). Effective human–AI collaboration, characterized by rational integration of AI advice with clinician expertise, has been shown to outperform either agent alone and is critical for enhancing diagnostic accuracy while preserving operator skills (158).

Importantly, the barriers to widespread AI adoption are not primarily economic. Rather, they lie in the technical, diagnostic, and behavioral hurdles discussed above. Overcoming them will be crucial to unlocking the full value of AI tools.

### 4.4 AI and personalized medicine

Looking ahead, the future of AI in endoscopy is closely aligned with the principles of personalized medicine. Machine learning–based decision-support systems could help individualize surveillance intervals by incorporating patient-specific risk factors, such as family history, previous polyp recurrence, and bowel preparation quality, thereby optimizing follow-up strategies and minimizing unnecessary procedures. However, while AI has clearly improved lesion detection and diagnostic accuracy in cancer screening and endoscopy, most studies rely on surrogate endpoints such as sensitivity, specificity, and ADR, rather than hard patient-centered outcomes like interval cancer rates or long-term prevention. In colonoscopy, AI-driven computer-aided detection systems have demonstrated increased ADR, but real-world data reveal gaps between improved diagnostic performance and actual clinical outcomes, emphasizing the need for further research in uncontrolled settings (151).

Umbrella reviews and systematic analyses confirm that, although AI algorithms consistently show high diagnostic accuracy across multiple cancer types, the overall quality of evidence is moderate to low, and there is a lack of studies directly linking AI-driven improvements to enhanced patient outcomes or survival (159). The medical literature highlights a critical gap between enhanced diagnostic performance and actual clinical benefit, underscoring the need for prospective, randomized studies to determine whether AI-driven improvements translate into improved patient outcomes, such as reduced interval cancer rates or long-term prevention.

### 4.5 Methodological limitations in AI validation

The challenge of generalizability for artificial intelligence models in gastrointestinal endoscopy is well-documented. Algorithms trained on curated datasets often demonstrate high diagnostic accuracy on selected images but may perform worse on real-world endoscopic data due to several factors: selection bias, limited variability in training images, differences in acquisition modalities, lack of external validation, and variable image quality such as motion blur, lighting differences, and suboptimal bowel preparation (160). These limitations impact all GI applications, from lesion detection in the colon to early neoplasia recognition in the upper GI tract.

Multicenter studies have shown that deep learning models for polyp detection and segmentation, when tested on out-of-sample datasets from different centers and devices, exhibit significant drops in performance, highlighting the need for improved generalizability and robustness to diverse clinical conditions (161). The American Society for Gastrointestinal Endoscopy (ASGE) consensus emphasizes that high-quality, multidimensional data and rigorous external validation are essential for reliable AI deployment in endoscopy, and that transparency and explainability are critical for clinical adoption (157). Real-world imaging conditions, such as motion artifacts, poor mucosal cleaning, and variable esophageal expansion, can substantially degrade the performance of AI systems trained on high-quality images, as demonstrated in Barrett’s neoplasia detection; incorporating diverse training data and domain-specific pretraining can mitigate some of these effects (162). To address these limitations, multicenter validation studies using diverse and representative datasets are essential to ensure that AI systems maintain high performance across different clinical settings, patient populations, and endoscopic equipment.

### 4.6 Biomedical challenges and AI

The translation of AI from experimental settings to real-world GI endoscopy has been strongly influenced by biomedical challenges related to data standardization, clinical relevance, and regulatory integration. A paradigmatic example is provided by the MICCAI Endoscopic Vision Challenges. Convolutional neural network–based polyp detection algorithms emerging from the MICCAI 2015 challenge have successfully transitioned into clinical practice, being integrated into commercial CADe systems for colonoscopy (163). These systems have demonstrated increased adenoma detection rates and reduced miss rates in prospective trials, largely due to real-time polyp flagging that enhances endoscopist performance and workflow efficiency. This successful clinical adoption was facilitated by the availability of standardized datasets, objective benchmarking, and clearly defined clinical endpoints, which supported regulatory approval and widespread implementation. In contrast, algorithms developed within the MICCAI 2018 challenge, focusing on multi-class disease classification and segmentation, achieved high accuracy and computational efficiency, enabling applications such as automated quality assessment, lesion characterization, and workflow optimization (164). While these methods have been incorporated into research platforms and selected commercial systems, their broader clinical adoption remains limited. Key biomedical challenges include limited generalizability across heterogeneous patient populations and endoscopic platforms, variability in disease presentation, and stringent regulatory requirements. Together, these experiences highlight how addressing biomedical complexity, rather than algorithmic performance alone, is critical for the successful advancement and integration of AI-driven endoscopic imaging into routine clinical practice. Several publicly available datasets now exist that provide essential resources for algorithm training, benchmarking, and validation; examples of these datasets, along with download links, are summarized in Table 4.

**TABLE 4:** Summary of several relevant datasets of artificial intelligence in gastrointestinal endoscopy.

Dataset	Finding/use case	Type	Download/access link
Kvasir	GI tract images (polyps, esophagitis, ulcers, etc.)	Image classification	https://datasets.simula.no/kvasir/ (datasets.simula.no)
Kvasir-SEG	Polyp images with segmentation masks	Polyp segmentation	https://datasets.simula.no/kvasir-seg/ (ITU)
Kvasir-capsule	Capsule endoscopy frames (millions)	Video/frames	https://datasets.simula.no/kvasir-capsule/ (ITU)
CVC-ClinicDB (CVC-612)	Polyp images with masks	Polyp detection/segmentation	https://polyp.grand-challenge.org/Databases/ (ITU)
ETIS-Larib Polyp DB	Polyp images with masks	Polyp detection/segmentation	https://polyp.grand-challenge.org/Databases/ (ITU)
PolypGen	Multi-center polyp frames (8,037)	Detection/segmentation	https://osf.io/84e7f/ (GitHub)
PolypDB	Multi-center polyp images (3,934) with multi-modal data	Polyp detection/segmentation	https://osf.io/pr7ms/ (arXiv)
REAL-Colon	∼2.7M full colonoscopy frames + annotations	Real-world colonoscopy videos	https://www.nature.com/articles/s41597-024-03359-0?utm_source=chatgpt.com
GastroVision	∼8,000 Multi-class GI endoscopy images	Multi-disease classification	https://osf.io/84e7f/ (GitHub)
GastroEndoNet	∼24,036 Endoscopic images (GERD + polyps)	Disease detection/classification	https://www.sciencedirect.com/science/article/pii/S235234092500304X
ERCPMP	Images/videos with polyp morphology/pathology	Polyp detection/classification	https://link.springer.com/article/10.1186/s13104-024-07062-6?utm_source=chatgpt.com
Auto-WCEBleedGen V1 and V2	Synthetic bleeding frames for WCE	Data synthesis and bleeding detection	https://www.techrxiv.org/users/715826/articles/719344-auto-wcebleedgen-version-v1-and-v2-challenge-datasets-and-evaluation?commit=82e4d3e1359d7ca23c2191f8913affd7a43ad01f
Capsule vision 2024—Multi-class abnormality classification for video capsule endoscopy	Multi-class abnormality classification in WCE	Video/frames classification	https://arxiv.org/abs/2408.04940

### 4.7 Clinical implementation considerations

Real-world adoption of AI-assisted endoscopy faces several key challenges. Cost and reimbursement policies are major barriers, as the initial investment in AI systems and uncertainty regarding reimbursement for AI-assisted procedures can limit uptake; robust cost-effectiveness data and clear payment models are needed to guide purchasing decisions and support sustainable implementation (157). Structured training is essential to ensure endoscopists understand AI capabilities, limitations, and error profiles, with professional societies emphasizing the need for education and competency development to maximize benefit and minimize risk (157).

Workflow integration requires careful planning to avoid disruption and ensure seamless incorporation of AI tools into existing clinical processes, including automated documentation and surveillance interval support (157). Tool selection guidance should be based on validated performance metrics and compatibility with local equipment and data infrastructure, as recommended by the European Society of Gastrointestinal Endoscopy and other expert groups (165). Evidence from multicenter studies highlights the importance of external validation to confirm generalizability and reliability of AI systems across diverse clinical settings, with prospective trials considered mandatory before widespread adoption (157).

Finally, the use of decision algorithms is advocated to balance diagnostic benefits with practical constraints, optimizing patient care by integrating AI outputs with clinical judgment and workflow realities (157). Addressing these barriers through collaborative efforts among clinicians, industry, and regulatory authorities is critical for the meaningful advancement of AI in endoscopy (157).

In conclusion, although the integration of AI into GI endoscopy still faces considerable clinical and technical challenges, its potential to enhance diagnostic accuracy, personalize patient care, and improve resource utilization is substantial. Future research must go beyond algorithm refinement and focus on understanding the dynamics of human–AI interaction. Rather than replacing endoscopists, AI should be viewed as an empowering tool supporting a more efficient, data-driven, and patient-centered approach to endoscopic practice.

## 5 Conclusion

AI is revolutionizing GI endoscopy, delivering real-time precision in detecting and classifying lesions while reducing unnecessary biopsies. From identifying dysplasia in Barrett’s esophagus to boosting adenoma detection rates in colonoscopy, AI-driven deep learning enhances accuracy and optimizes surveillance strategies. By integrating imaging with clinical and molecular biomarkers, AI paves the way for smarter, more efficient diagnostics. As its role expands, overcoming challenges like standardization and validation will be key to unlocking its full potential in routine practice. The future of endoscopy is here powered by AI, transforming diagnosis and decision-making like never before.

## References

[1] Ali H Muzammil M Dahiya D Ali F Yasin S Hanif W Artificial intelligence in gastrointestinal endoscopy: a comprehensive review. *Ann Gastroenterol.* (2024) 37:133–41. 10.20524/aog.2024.0861 38481787 PMC10927620

[2] East J Vleugels J Roelandt P Bhandari P Bisschops R Dekker E Advanced endoscopic imaging: European society of gastrointestinal *Endoscopy* (ESGE) technology review. *Endoscopy.* (2016) 48:1029–45. 10.1055/s-0042-118087 27711949

[3] Hancock S Bowman E Prabakaran J Benson M Agni R Pfau P Use of i-scan endoscopic image enhancement technology in clinical practice to assist in diagnostic and therapeutic endoscopy: a case series and review of the literature. *Diagn Ther Endosc.* (2012) 2012:193570. 10.1155/2012/193570 23243384 PMC3518095

[4] Yang Q Liu Z Sun H Jiao F Zhang B Chen JA. narrative review: narrow-band imaging endoscopic classifications. *Quant Imaging Med Surg.* (2023) 13:1138–63. 10.21037/qims-22-728 36819279 PMC9929402

[5] Subramanian V Ragunath K. Advanced endoscopic imaging: a review of commercially available technologies. *Clin Gastroenterol Hepatol.* (2014) 12:368–76.e1. 10.1016/j.cgh.2013.06.015 23811245

[6] Boeriu A Boeriu C Drasovean S Pascarenco O Mocan S Stoian M Narrow-band imaging with magnifying endoscopy for the evaluation of gastrointestinal lesions. *World J Gastrointest Endosc.* (2015) 7:110–20. 10.4253/wjge.v7.i2.110 25685267 PMC4325307

[7] Shinozaki S Osawa H Hayashi Y Lefor A Yamamoto H. Linked color imaging for the detection of early gastrointestinal neoplasms. *Therap Adv Gastroenterol.* (2019) 12:1756284819885246. 10.1177/1756284819885246 31700545 PMC6826899

[8] Yashima K Onoyama T Kurumi H Takeda Y Yoshida A Kawaguchi K Current status and future perspective of linked color imaging for gastric cancer screening: a literature review. *J Gastroenterol.* (2023) 58:1–13. 10.1007/s00535-022-01934-z 36287268 PMC9825522

[9] Min M Sun X Bai J Zhang Q Yang X Guo Q Diagnostic accuracy of linked colour imaging versus white light imaging for early gastric cancers: a prospective, multicentre, randomized controlled trial study. *Ann Med.* (2022) 54:3306–14. 10.1080/07853890.2022.2147991 36411585 PMC9704855

[10] Pilonis N Januszewicz W di Pietro M. Confocal laser endomicroscopy in gastro-intestinal endoscopy: technical aspects and clinical applications. *Transl Gastroenterol Hepatol.* (2022) 7:7. 10.21037/tgh.2020.04.02 35243116 PMC8826043

[11] Wang K Carr-Locke D Singh S Neumann H Bertani H Galmiche J Use of probe-based confocal laser endomicroscopy (pCLE) in gastrointestinal applications. A consensus report based on clinical evidence. *United Eur Gastroenterol J.* (2015) 3:230–54. 10.1177/2050640614566066 26137298 PMC4480534

[12] Tang Y Anandasabapathy S Richards-Kortum R. Advances in optical gastrointestinal endoscopy: a technical review. *Mol Oncol.* (2021) 15:2580–99. 10.1002/1878-0261.12792 32915503 PMC8486567

[13] Lim S. Advances and challenges in gastrointestinal endoscopy: a comprehensive review. *J Innov Med Technol.* (2023) 1:10–4. 10.61940/jimt.230008

[14] Rey J. As how artificial intelligence is revolutionizing endoscopy. *Clin Endosc.* (2024) 57:302–8. 10.5946/ce.2023.230 38454543 PMC11133999

[15] Mori Y Misawa M. Quality assessment in endoscopy artificial intelligence in endoscopy. *Best Pract Res Clin Gastroenterol.* (2025) 76:102006. 10.1016/j.bpg.2025.102006 40610178

[16] Hassan C Spadaccini M Mori Y Foroutan F Facciorusso A Gkolfakis P Real-time computer-aided detection of colorectal neoplasia during colonoscopy : a systematic review and meta-analysis. *Ann Intern Med.* (2023) 176:1209–20. 10.7326/M22-3678 37639719

[17] Iacucci M Santacroce G Yasuharu M Ghosh S. Artificial intelligence-driven personalized medicine: transforming clinical practice in inflammatory bowel disease. *Gastroenterology.* (2025) 169:416–31. 10.1053/j.gastro.2025.03.005 40074186

[18] Iacucci M Jeffery L Acharjee A Grisan E Buda A Nardone O Computer-aided imaging analysis of probe-based confocal laser endomicroscopy with molecular labeling and gene expression identifies markers of response to biological therapy in IBD patients: the endo-omics study. *Inflamm Bowel Dis.* (2023) 29:1409–20. 10.1093/ibd/izac233 36378498 PMC10472745

[19] Chen J Liu S Gao T. Research progress on endoscopic multimodal optical imaging. *Optics Laser Technol.* (2025) 191:113355. 10.1016/j.optlastec.2025.113355

[20] Mascarenhas M Mendes F Martins M Ribeiro T Afonso J Cardoso P Explainable AI in digestive healthcare and gastrointestinal endoscopy. *J Clin Med.* (2025) 14:549. 10.3390/jcm14020549 39860554 PMC11765989

[21] Phillips H Fetzer J Bhattarai S Perneth S Codipilly D Ebner D Computer-assisted classification of the squamocolumnar junction. *Gastrointest Endosc.* (2025) 102:245–51. 10.1016/j.gie.2025.01.020 39832556

[22] Barchi A Dell’Anna G Massimino L Mandarino F Vespa E Viale E Unraveling the pathogenesis of Barrett’s esophagus and esophageal adenocarcinoma: the era. *Front Oncol.* (2024) 14:1458138. 10.3389/fonc.2024.1458138 39950103 PMC11821489

[23] Visrodia K Singh S Krishnamoorthi R Ahlquist D Wang K Iyer P Magnitude of missed esophageal adenocarcinoma after barrett’s esophagus diagnosis: a systematic review and meta-analysis. *Gastroenterology.* (2016) 150:599–607.e7. 10.1053/j.gastro.2015.11.040 26619962 PMC4919075

[24] Ebigbo A Messmann H Lee S. Artificial intelligence applications in image-based diagnosis of early esophageal and gastric neoplasms. *Gastroenterology.* (2025) 169:396–415.e2. 10.1053/j.gastro.2025.01.253 40043857

[25] de Groof A Struyvenberg M van der Putten J van der Sommen F Fockens K Curvers W Deep-Learning system detects neoplasia in patients with barrett’s esophagus with higher accuracy than endoscopists in a multistep training and validation study with benchmarking. *Gastroenterology.* (2020) 158:915–29.e4. 10.1053/j.gastro.2019.11.030 31759929

[26] Hamade N Sharma P. Artificial intelligence in Barrett’s Esophagus. *Clin Med Insights Gastroenterol.* (2021) 14:26317745211049964. 10.1177/26317745211049964 34671724 PMC8521738

[27] de Groof A Struyvenberg M Fockens K van der Putten J van der Sommen F Boers T Deep learning algorithm detection of Barrett’s neoplasia with high accuracy during live endoscopic procedures: a pilot study (with video). *Gastrointest Endosc.* (2020) 91:1242–50. 10.1016/j.gie.2019.12.048 31926965

[28] Ebigbo A Mendel R Probst A Manzeneder J Prinz F de Souza L Real-time use of artificial intelligence in the evaluation of cancer in Barrett’s oesophagus. *Gut.* (2020) 69:615–6. 10.1136/gutjnl-2019-319460 31541004 PMC7063447

[29] Abdelrahim M Saiko M Maeda N Hossain E Alkandari A Subramaniam S Development and validation of artificial neural networks model for detection of Barrett’s neoplasia: a multicenter pragmatic nonrandomized trial (with video). *Gastrointest Endosc.* (2023) 97:422–34. 10.1016/j.gie.2022.10.031 36283443

[30] Hussein M González-Bueno Puyal J Lines D Sehgal V Toth D Ahmad O A new artificial intelligence system successfully detects and localises early neoplasia in Barrett’s esophagus by using convolutional neural networks. *United Eur Gastroenterol J.* (2022) 10:528–37. 10.1002/ueg2.12233 35521666 PMC9278593

[31] Schmitz R Kather J. Artificial intelligence in Barrett’s oesophagus and the need for shared and combined data. *United Eur Gastroenterol J.* (2022) 10:525–7. 10.1002/ueg2.12260 35704382 PMC9278571

[32] Jong M de Groof A. Advancement of artificial intelligence systems for surveillance endoscopy of Barrett’s esophagus. *Dig Liver Dis.* (2024) 56:1126–30. 10.1016/j.dld.2023.11.038 38071181

[33] Fockens K Jong M Jukema J Boers T Kusters C van der Putten J A deep learning system for detection of early Barrett’s neoplasia: a model development and validation study. *Lancet Digit Health.* (2023) 5:e905–16. 10.1016/S2589-7500(23)00199-1 38000874

[34] Jong M Jaspers T Kusters C Jukema J van Eijck van Heslinga RAH Fockens KN Challenges in implementing endoscopic artificial intelligence: the impact of real-world imaging conditions on Barrett’s Neoplasia detection. *United Eur Gastroenterol J.* (2025) 13:929–37. 10.1002/ueg2.12760 40116287 PMC12269737

[35] Meinikheim M Mendel R Palm C Probst A Muzalyova A Scheppach M Influence of artificial intelligence on the diagnostic performance of endoscopists in the assessment of Barrett’s esophagus: a tandem randomized and video trial. *Endoscopy.* (2024) 56:641–9. 10.1055/a-2296-5696 38547927

[36] Sharma P Hassan C. Artificial intelligence and deep learning for upper gastrointestinal neoplasia. *Gastroenterology.* (2022) 162:1056–66. 10.1053/j.gastro.2021.11.040 34902362

[37] Hashimoto R Requa J Dao T Ninh A Tran E Mai D Artificial intelligence using convolutional neural networks for real-time detection of early esophageal neoplasia in Barrett’s esophagus (with video). *Gastrointest Endosc.* (2020) 91:1264–71.e1. 10.1016/j.gie.2019.12.049 31930967

[38] Hussein M Lines D González-Bueno Puyal J Kader R Bowman N Sehgal V Computer-aided characterization of early cancer in Barrett’s esophagus on i-scan magnification imaging: a multicenter international study. *Gastrointest Endosc.* (2023) 97:646–54. 10.1016/j.gie.2022.11.020 36460087 PMC10590905

[39] Jukema J Kusters C Jong M Fockens K Boers T van der Putten J Computer-aided diagnosis improves characterization of Barrett’s neoplasia by general endoscopists (with video). *Gastrointest Endosc.* (2024) 100:616–25.e8. 10.1016/j.gie.2024.04.013 38636819

[40] Gupta S Mandarino F Shahidi N Hourigan L Messmann H Wallace M Can optical evaluation distinguish between T1a and T1b esophageal adenocarcinoma: an international expert interobserver agreement study. *Endoscopy.* (2024) 57:200–7. 10.1055/a-2399-1401 39168143

[41] Ebigbo A Mendel R Rückert T Schuster L Probst A Manzeneder J Endoscopic prediction of submucosal invasion in Barrett’s cancer with the use of artificial intelligence: a pilot study. *Endoscopy.* (2021) 53:878–83. 10.1055/a-1311-8570 33197942

[42] Morita F Bernardo W Ide E Rocha R Aquino J Minata M Narrow band imaging versus lugol chromoendoscopy to diagnose squamous cell carcinoma of the esophagus: a systematic review and meta-analysis. *BMC Cancer.* (2017) 17:54. 10.1186/s12885-016-3011-9 28086818 PMC5237308

[43] Arribas J Antonelli G Frazzoni L Fuccio L Ebigbo A van der Sommen F Standalone performance of artificial intelligence for upper GI neoplasia: a meta-analysis. *Gut.* (2021): 10.1136/gutjnl-2020-321922 Online ahead of print. 33127833

[44] Ohmori M Ishihara R Aoyama K Nakagawa K Iwagami H Matsuura N Endoscopic detection and differentiation of esophageal lesions using a deep neural network. *Gastrointest Endosc.* (2020) 91:301–9.e1. 10.1016/j.gie.2019.09.034 31585124

[45] Guo L Xiao X Wu C Zeng X Zhang Y Du J Real-time automated diagnosis of precancerous lesions and early esophageal squamous cell carcinoma using a deep learning model (with videos). *Gastrointest Endosc.* (2020) 91:41–51. 10.1016/j.gie.2019.08.018 31445040

[46] Cai S Li B Tan W Niu X Yu H Yao L Using a deep learning system in endoscopy for screening of early esophageal squamous cell carcinoma (with video). *Gastrointest Endosc.* (2019) 90:745–53.e2. 10.1016/j.gie.2019.06.044 31302091

[47] Waki K Ishihara R Kato Y Shoji A Inoue T Matsueda K Usefulness of an artificial intelligence system for the detection of esophageal squamous cell carcinoma evaluated with videos simulating overlooking situation. *Dig Endosc.* (2021) 33:1101–9. 10.1111/den.13934 33502046

[48] Shiroma S Yoshio T Kato Y Horie Y Namikawa K Tokai Y Ability of artificial intelligence to detect T1 esophageal squamous cell carcinoma from endoscopic videos and the effects of real-time assistance. *Sci Rep.* (2021) 11:7759. 10.1038/s41598-021-87405-6 33833355 PMC8032773

[49] Yuan X Liu W Lin Y Deng Q Gao Y Wan L Effect of an artificial intelligence-assisted system on endoscopic diagnosis of superficial oesophageal squamous cell carcinoma and precancerous lesions: a multicentre, tandem, double-blind, randomised controlled trial. *Lancet Gastroenterol Hepatol.* (2024) 9:34–44. 10.1016/S2468-1253(23)00276-5 37952555

[50] Hu B. Exploring artificial intelligence-assisted diagnosis of esophageal squamous cell carcinoma: insights from a clinical trial. *Endoscopy.* (2025) 57:218–9. 10.1055/a-2466-6331 39608407

[51] Li S Zhang L Cai Y Zhou X Fu X Song Y Deep learning assists detection of esophageal cancer and precursor lesions in a prospective, randomized controlled study. *Sci Transl Med.* (2024) 16:eadk5395. 10.1126/scitranslmed.adk5395 38630847

[52] Zhou Y Liu R Gong H Yuan X Hu B Huang Z. Multimodal artificial intelligence system for detecting a small esophageal high-grade squamous intraepithelial neoplasia: a case report. *World J Gastrointest Endosc.* (2025) 17:101233. 10.4253/wjge.v17.i1.101233 39850915 PMC11752473

[53] Shimamoto Y Ishihara R Kato Y Shoji A Inoue T Matsueda K Real-time assessment of video images for esophageal squamous cell carcinoma invasion depth using artificial intelligence. *J Gastroenterol.* (2020) 55:1037–45. 10.1007/s00535-020-01716-5 32778959

[54] Eshmuratov A Nah J Kim N Lee H Lee H Lee B The correlation of endoscopic and histological diagnosis of gastric atrophy. *Dig Dis Sci.* (2010) 55:1364–75. 10.1007/s10620-009-0891-4 19629687

[55] Honing J Keith Tan W Dieninyte E O’Donovan M Brosens L Weusten B Adequacy of endoscopic recognition and surveillance of gastric intestinal metaplasia and atrophic gastritis: a multicentre retrospective study in low incidence countries. *PLoS One.* (2023) 18:e0287587. 10.1371/journal.pone.0287587 37352223 PMC10289343

[56] Guimarães P Keller A Fehlmann T Lammert F Casper M. Deep-learning based detection of gastric precancerous conditions. *Gut.* (2020) 69:4–6. 10.1136/gutjnl-2019-319347 31375599

[57] Xu M Zhou W Wu L Zhang J Wang J Mu G Artificial intelligence in the diagnosis of gastric precancerous conditions by image-enhanced endoscopy: a multicenter, diagnostic study (with video). *Gastrointest Endosc.* (2021) 94:540–8.e4. 10.1016/j.gie.2021.03.013 33722576

[58] Xu Z Li Y Su P Zhong Z Zeng Z Chen M Artificial intelligence system improves the quality of digestive endoscopy: a prospective pretest and post-test single-center clinical trial. *Dig Liver Dis.* (2025) 57:1830–7. 10.1016/j.dld.2025.04.029 40345942

[59] Wu L Zhou W Wan X Zhang J Shen L Hu S A deep neural network improves endoscopic detection of early gastric cancer without blind spots. *Endoscopy.* (2019) 51:522–31. 10.1055/a-0855-3532 30861533

[60] Ling T Wu L Fu Y Xu Q An P Zhang J A deep learning-based system for identifying differentiation status and delineating the margins of early gastric cancer in magnifying narrow-band imaging endoscopy. *Endoscopy.* (2020) 53:469–77. 10.1055/a-1229-0920 32725617

[61] Wu L Wang J He X Zhu Y Jiang X Chen Y Deep learning system compared with expert endoscopists in predicting early gastric cancer and its invasion depth and differentiation status (with videos). *Gastrointest Endosc.* (2022) 95:92–104.e3. 10.1016/j.gie.2021.06.033 34245752

[62] Wu L Shang R Sharma P Zhou W Liu J Yao L Effect of a deep learning-based system on the miss rate of gastric neoplasms during upper gastrointestinal endoscopy: a single-centre, tandem, randomised controlled trial. *Lancet Gastroenterol Hepatol.* (2021) 6:700–8. 10.1016/S2468-1253(21)00216-8 34297944

[63] Kusano C. Artificial intelligence for gastric cancer: can we make further progress? *Endoscopy.* (2021) 53:1208–9. 10.1055/a-1471-3474 34318448

[64] Ahmad O. Early detection of gastric neoplasia: is artificial intelligence the solution? *Lancet Gastroenterol Hepatol.* (2021) 6:678–9. 10.1016/S2468-1253(21)00254-5 34297943

[65] Luo H Xu G Li C He L Luo L Wang Z Real-time artificial intelligence for detection of upper gastrointestinal cancer by endoscopy: a multicentre, case-control, diagnostic study. *Lancet Oncol.* (2019) 20:1645–54. 10.1016/S1470-2045(19)30637-0 31591062

[66] Choi J Kim S Im J Kim J Jung H Song I. Comparison of endoscopic ultrasonography and conventional endoscopy for prediction of depth of tumor invasion in early gastric cancer. *Endoscopy.* (2010) 42:705–13. 10.1055/s-0030-1255617 20652857

[67] Zhu Y Wang Q Xu M Zhang Z Cheng J Zhong Y Application of convolutional neural network in the diagnosis of the invasion depth of gastric cancer based on conventional endoscopy. *Gastrointest Endosc.* (2019) 89:806–15.e1. 10.1016/j.gie.2018.11.011 30452913

[68] Chen T Kuo C Lee C Yeh T Lan J Huang S. Artificial intelligence model for a distinction between early-stage gastric cancer invasive depth T1a and T1b. *J Cancer.* (2024) 15:3085–94. 10.7150/jca.94772 38706899 PMC11064248

[69] Chiang T Hsu Y Chen M Chen Y Cheng H Chen M A rural-to-center artificial intelligence model for diagnosing *Helicobacter* pylori infection and premalignant gastric conditions using endoscopy images captured in routine practice. *Endoscopy.* (2025): 10.1055/a-2721-6552 Online ahead of print. 41082919 PMC13063403

[70] Nakashima H Kawahira H Kawachi H Sakaki N. Endoscopic three-categorical diagnosis of *Helicobacter* pylori infection using linked color imaging and deep learning: a single-center prospective study (with video). *Gastric Cancer.* (2020) 23:1033–40. 10.1007/s10120-020-01077-1 32382973

[71] De Palma F D’Argenio V Pol J Kroemer G Maiuri M Salvatore F. The molecular hallmarks of the serrated pathway in colorectal cancer. *Cancers.* (2019) 11:1017. 10.3390/cancers11071017 31330830 PMC6678087

[72] Nguyen H Duong H. The molecular characteristics of colorectal cancer: implications for diagnosis and therapy. *Oncol Lett.* (2018) 16:9–18. 10.3892/ol.2018.8679 29928381 PMC6006272

[73] Wang J Xu G Hu X Li W Yao N Han F The histologic features, molecular features, detection and management of serrated polyps: a review. *Front Oncol.* (2024) 14:1356250. 10.3389/fonc.2024.1356250 38515581 PMC10955069

[74] East J Vieth M Rex D. Serrated lesions in colorectal cancer screening: detection, resection, pathology and surveillance. *Gut.* (2015) 64:991–1000. 10.1136/gutjnl-2014-309041 25748647

[75] Ferlitsch M Hassan C Bisschops R Bhandari P Dinis-Ribeiro M Risio M Colorectal polypectomy and endoscopic mucosal resection: European society of gastrointestinal *endoscopy* (ESGE) guideline - update 2024. *Endoscopy.* (2024) 56:516–45. 10.1055/a-2304-3219 38670139

[76] Hassan C Balsamo G Lorenzetti R Zullo A Antonelli G. Artificial intelligence allows leaving-in-situ colorectal polyps. *Clin Gastroenterol Hepatol.* (2022) 20:2505–13.e4. 10.1016/j.cgh.2022.04.045 35835342

[77] Tischendorf J Gross S Winograd R Hecker H Auer R Behrens A Computer-aided classification of colorectal polyps based on vascular patterns: a pilot study. *Endoscopy.* (2010) 42:203–7. 10.1055/s-0029-1243861 20101564

[78] Tamaki T Yoshimuta J Kawakami M Raytchev B Kaneda K Yoshida S Computer-aided colorectal tumor classification in NBI endoscopy using local features. *Med Image Anal.* (2013) 17:78–100. 10.1016/j.media.2012.08.003 23085199

[79] Hirakawa T Tamaki T Raytchev B Kaneda K Koide T Kominami Y SVM-MRF segmentation of colorectal NBI endoscopic images. *Annu Int Conf IEEE Eng Med Biol Soc.* (2014) 2014:4739–42. 10.1109/EMBC.2014.6944683 25571051

[80] Häfner M Tamaki T Tanaka S Uhl A Wimmer G Yoshida S. Local fractal dimension based approaches for colonic polyp classification. *Med Image Anal.* (2015) 26:92–107. 10.1016/j.media.2015.08.007 26385078

[81] Wimmer G Tamaki T Tischendorf J Häfner M Yoshida S Tanaka S Directional wavelet based features for colonic polyp classification. *Med Image Anal.* (2016) 31:16–36. 10.1016/j.media.2016.02.001 26948110

[82] Okamoto T Koide T Sugi K Shimizu T Anh-Tuan Hoang, Tamaki T Image segmentation of pyramid style identifier based on support vector machine for colorectal endoscopic images. *Annu Int Conf IEEE Eng Med Biol Soc.* (2015) 2015:2997–3000. 10.1109/EMBC.2015.7319022 26736922

[83] Chen P Lin M Lai M Lin J Lu H Tseng V. Accurate classification of diminutive colorectal polyps using computer-aided analysis. *Gastroenterology.* (2018) 154:568–75. 10.1053/j.gastro.2017.10.010 29042219

[84] Rex D Kahi C O’Brien M Levin T Pohl H Rastogi A The American society for gastrointestinal endoscopy PIVI (Preservation and incorporation of valuable endoscopic innovations) on real-time endoscopic assessment of the histology of diminutive colorectal polyps. *Gastrointest Endosc.* (2011) 73:419–22. 10.1016/j.gie.2011.01.023 21353837

[85] Byrne M Chapados N Soudan F Oertel C Linares Pérez M Kelly R Real-time differentiation of adenomatous and hyperplastic diminutive colorectal polyps during analysis of unaltered videos of standard colonoscopy using a deep learning model. *Gut.* (2019) 68:94–100. 10.1136/gutjnl-2017-314547 29066576 PMC6839831

[86] Aihara H Saito S Inomata H Ide D Tamai N Ohya T Computer-aided diagnosis of neoplastic colorectal lesions using ‘real-time’ numerical color analysis during autofluorescence endoscopy. *Eur J Gastroenterol Hepatol.* (2013) 25:488–94. 10.1097/MEG.0b013e32835c6d9a 23249604

[87] Mori Y Kudo S Misawa M Saito Y Ikematsu H Hotta K Real-time use of artificial intelligence in identification of diminutive polyps during colonoscopy: a prospective study. *Ann Intern Med.* (2018) 169:357–66. 10.7326/M18-0249 30105375

[88] Kuiper T Alderlieste Y Tytgat K Vlug M Nabuurs J Bastiaansen B Automatic optical diagnosis of small colorectal lesions by laser-induced autofluorescence. *Endoscopy.* (2015) 47:56–62. 10.1055/s-0034-1378112 25264763

[89] Rath T Tontini G Vieth M Nägel A Neurath M Neumann H. In vivo real-time assessment of colorectal polyp histology using an optical biopsy forceps system based on laser-induced fluorescence spectroscopy. *Endoscopy.* (2016) 48:557–62. 10.1055/s-0042-102251 27009081

[90] Shimizu T Sasaki Y Ito K Matsuzaka M Sakuraba H Fukuda SA. trial deep learning-based model for four-class histologic classification of colonic tumor from narrow band imaging. *Sci Rep.* (2023) 13:7510. 10.1038/s41598-023-34750-3 37161081 PMC10169849

[91] Houwen B Hazewinkel Y Giotis I Vleugels J Mostafavi N van Putten P Computer-aided diagnosis for optical diagnosis of diminutive colorectal polyps including sessile serrated lesions: a real-time comparison with screening endoscopists. *Endoscopy.* (2023) 55:756–65. 10.1055/a-2009-3990 36623839 PMC10374350

[92] Chan I Wong P Yan T Multi-modal machine learning in gastrointestinal endoscopy: A review. *In Proceedings of the 2024 11th International Conference on Biomedical and Bioinformatics Engineering.* New York, NY: Association for Computing Machinery (2025). p. 10–7. 10.1145/3707127.3707129

[93] Spada C Salvi D Ferrari C Hassan C Barbaro F Belluardo N A comprehensive RCT in screening, surveillance, and diagnostic AI-assisted colonoscopies (ACCENDO-Colo study). *Dig Liver Dis.* (2025) 57:762–9. 10.1016/j.dld.2024.12.023 39814659

[94] Repici A Spadaccini M Antonelli G Correale L Maselli R Galtieri P Artificial intelligence and colonoscopy experience: lessons from two randomised trials. *Gut.* (2022) 71:757–65. 10.1136/gutjnl-2021-324471 34187845

[95] Mangas-Sanjuan C de-Castro L Cubiella J Díez-Redondo P Suárez A Pellisé M Role of artificial intelligence in colonoscopy detection of advanced neoplasias : a randomized trial. *Ann Intern Med.* (2023) 176:1145–52. 10.7326/M22-2619 37639723

[96] Hiratsuka Y Hisabe T Ohtsu K Yasaka T Takeda K Miyaoka M Evaluation of artificial intelligence: computer-aided detection of colorectal polyps. *J Anus Rectum Colon.* (2025) 9:79–87. 10.23922/jarc.2024-057 39882222 PMC11772790

[97] Soleymanjahi S Huebner J Elmansy L Rajashekar N Lüdtke N Paracha R Artificial intelligence-assisted colonoscopy for polyp detection : a systematic review and meta-analysis. *Ann Intern Med.* (2024) 177:1652–63. 10.7326/ANNALS-24-00981 39531400

[98] Maida M Marasco G Maas M Ramai D Spadaccini M Sinagra E Effectiveness of artificial intelligence assisted colonoscopy on adenoma and polyp miss rate: a meta-analysis of tandem RCTs. *Dig Liver Dis.* (2025) 57:169–75. 10.1016/j.dld.2024.09.003 39322447

[99] Spadaccini M Hassan C Mori Y Massimi D Correale L Facciorusso A Variability in computer-aided detection effect on adenoma detection rate in randomized controlled trials: a meta-regression analysis. *Dig Liver Dis.* (2025) 57:1141–8. 10.1016/j.dld.2025.01.192 39924430

[100] Ortiz O Daca-Alvarez M Rivero-Sanchez L Gimeno-Garcia A Carrillo-Palau M Alvarez V An artificial intelligence-assisted system versus white light endoscopy alone for adenoma detection in individuals with Lynch syndrome (TIMELY): an international, multicentre, randomised controlled trial. *Lancet Gastroenterol Hepatol.* (2024) 9:802–10. 10.1016/S2468-1253(24)00187-0 39033774

[101] Zhao S Wang S Pan P Xia T Chang X Yang X Magnitude, risk factors, and factors associated with adenoma miss rate of tandem colonoscopy: a systematic review and meta-analysis. *Gastroenterology.* (2019) 156:1661–74.e11. 10.1053/j.gastro.2019.01.260 30738046

[102] Ali S Jha D Ghatwary N Realdon S Cannizzaro R Salem O A multi-centre polyp detection and segmentation dataset for generalisability assessment. *Sci Data.* (2023) 10:75. 10.1038/s41597-023-01981-y 36746950 PMC9902556

[103] Testoni S Albertini Petroni G Annunziata M Dell’Anna G Puricelli M Delogu C Artificial intelligence in inflammatory bowel disease endoscopy. *Diagnostics.* (2025) 15:905. 10.3390/diagnostics15070905 40218255 PMC11988936

[104] Turner D Ricciuto A Lewis A D’Amico F Dhaliwal J Griffiths A STRIDE-II: an update on the Selecting therapeutic targets in inflammatory bowel disease (STRIDE) Initiative of the international organization for the study of IBD (IOIBD): determining therapeutic goals for treat-to-target strategies in IBD. *Gastroenterology.* (2021) 160:1570–83. 10.1053/j.gastro.2020.12.031 33359090

[105] Katsanos K Papamichael K Christodoulou D Cheifetz A. Histological healing beyond endoscopic healing in ulcerative colitis: shall we target the ultra-deep remission? *Dig Liver Dis.* (2017) 49:1332–3. 10.1016/j.dld.2017.08.043 28964677

[106] Pabla B Schwartz D. Assessing severity of disease in patients with ulcerative colitis. *Gastroenterol Clin North Am.* (2020) 49:671–88. 10.1016/j.gtc.2020.08.003 33121688 PMC7510557

[107] Neumann H Vieth M Günther C Neufert C Kiesslich R Grauer M Virtual chromoendoscopy for prediction of severity and disease extent in patients with inflammatory bowel disease: a randomized controlled study. *Inflamm Bowel Dis.* (2013) 19:1935–42. 10.1097/MIB.0b013e318290550e 23839228

[108] Ozawa T Ishihara S Fujishiro M Saito H Kumagai Y Shichijo S Novel computer-assisted diagnosis system for endoscopic disease activity in patients with ulcerative colitis. *Gastrointest Endosc.* (2019) 89:416–21.e1. 10.1016/j.gie.2018.10.020 30367878

[109] Stidham R Liu W Bishu S Rice M Higgins P Zhu J Performance of a deep learning model vs human reviewers in grading endoscopic disease severity of patients with ulcerative colitis. *JAMA Netw Open.* (2019) 2:e193963. 10.1001/jamanetworkopen.2019.3963 31099869 PMC6537821

[110] Takenaka K Ohtsuka K Fujii T Negi M Suzuki K Shimizu H Development and validation of a deep neural network for accurate evaluation of endoscopic images from patients with ulcerative colitis. *Gastroenterology.* (2020) 158:2150–7. 10.1053/j.gastro.2020.02.012 32060000

[111] Takenaka K Fujii T Kawamoto A Suzuki K Shimizu H Maeyashiki C Deep neural network for video colonoscopy of ulcerative colitis: a cross-sectional study. *Lancet Gastroenterol Hepatol.* (2022) 7:230–7. 10.1016/S2468-1253(21)00372-1 34856196

[112] Yao H Najarian K Gryak J Bishu S Rice M Waljee A Fully automated endoscopic disease activity assessment in ulcerative colitis. *Gastrointest Endosc.* (2021) 93:728–36.e1. 10.1016/j.gie.2020.08.011 32810479

[113] Rimondi A Gottlieb K Despott E Iacucci M Murino A Tontini G. Can artificial intelligence replace endoscopists when assessing mucosal healing in ulcerative colitis? A systematic review and diagnostic test accuracy meta-analysis. *Dig Liver Dis.* (2024) 56:1164–72. 10.1016/j.dld.2023.11.005 38057218

[114] Lv B Ma L Shi Y Tao T Shi Y. A systematic review and meta-analysis of artificial intelligence-diagnosed endoscopic remission in ulcerative colitis. *iScience.* (2023) 26:108120. 10.1016/j.isci.2023.108120 37867944 PMC10585391

[115] Jahagirdar V Bapaye J Chandan S Ponnada S Kochhar G Navaneethan U Diagnostic accuracy of convolutional neural network-based machine learning algorithms in endoscopic severity prediction of ulcerative colitis: a systematic review and meta-analysis. *Gastrointest Endosc.* (2023) 98:145–54.e8. 10.1016/j.gie.2023.04.2074 37094691

[116] Takabayashi K Kobayashi T Matsuoka K Levesque B Kawamura T Tanaka K Artificial intelligence quantifying endoscopic severity of ulcerative colitis in gradation scale. *Dig Endosc.* (2024) 36:582–90. 10.1111/den.14677 37690125 PMC12136255

[117] Fan Y Mu R Xu H Xie C Zhang Y Liu L Novel deep learning-based computer-aided diagnosis system for predicting inflammatory activity in ulcerative colitis. *Gastrointest Endosc.* (2023) 97:335–46. 10.1016/j.gie.2022.08.015 35985375

[118] Stidham R Cai L Cheng S Rajaei F Hiatt T Wittrup E Using computer vision to improve endoscopic disease quantification in therapeutic clinical trials of ulcerative colitis. *Gastroenterology.* (2024) 166:155–67.e2. 10.1053/j.gastro.2023.09.049 37832924

[119] Gottlieb K Requa J Karnes W Chandra Gudivada R Shen J Rael E Central reading of ulcerative colitis clinical trial videos using neural networks. *Gastroenterology.* (2021) 160:710–9.e2. 10.1053/j.gastro.2020.10.024 33098883

[120] Iacucci M Zammarchi I Santacroce G Kolawole B Chaudhari U Del Amor R A Novel switching of artificial intelligence to generate simultaneously multimodal images to assess inflammation and predict outcomes in ulcerative colitis-(With Video). *Dig Endosc.* (2025) 37:1078–88. 10.1111/den.15067 40518924 PMC12511918

[121] Iacucci M Cannatelli R Parigi T Nardone O Tontini G Labarile N A virtual chromoendoscopy artificial intelligence system to detect endoscopic and histologic activity/remission and predict clinical outcomes in ulcerative colitis. *Endoscopy.* (2023) 55:332–41. 10.1055/a-1960-3645 36228649 PMC10060056

[122] Puga-Tejada M Majumder S Maeda Y Zammarchi I Ditonno I Santacroce G Artificial intelligence-enabled histology exhibits comparable accuracy to pathologists in assessing histological remission in ulcerative colitis: a systematic review, meta-analysis, and meta-regression. *J Crohns Colitis.* (2025) 19:jjae198. 10.1093/ecco-jcc/jjae198 39742395 PMC11724188

[123] Ogata N Ohtsuka K Ogawa M Maeda Y Ishida F Kudo S. Image-enhanced capsule endoscopy improves the identification of small intestinal lesions. *Diagnostics.* (2021) 11:2122. 10.3390/diagnostics11112122 34829469 PMC8621083

[124] Klang E Barash Y Margalit R Soffer S Shimon O Albshesh A Deep learning algorithms for automated detection of Crohn’s disease ulcers by video capsule endoscopy. *Gastrointest Endosc.* (2020) 91:606–13.e2. 10.1016/j.gie.2019.11.012 31743689

[125] Majtner T Brodersen J Herp J Kjeldsen J Halling M Jensen MD. A deep learning framework for autonomous detection and classification of Crohn’s disease lesions in the small bowel and colon with capsule endoscopy. *Endosc Int Open.* (2021) 9:E1361–70. 10.1055/a-1507-4980 34466360 PMC8367448

[126] Kellerman R Bleiweiss A Samuel S Margalit-Yehuda R Aflalo E Barzilay O Spatiotemporal analysis of small bowel capsule endoscopy videos for outcomes prediction in Crohn’s disease. *Therap Adv Gastroenterol.* (2023) 16:17562848231172556. 10.1177/17562848231172556 37440929 PMC10333642

[127] Brodersen J Jensen M Leenhardt R Kjeldsen J Histace A Knudsen T Artificial intelligence-assisted analysis of pan-enteric capsule endoscopy in patients with suspected Crohn’s disease: a study on diagnostic performance. *J Crohns Colitis.* (2024) 18:75–81. 10.1093/ecco-jcc/jjad131 37527554

[128] Fanizza J Bencardino S Allocca M Furfaro F Zilli A Parigi T Inflammatory bowel disease and colorectal cancer. *Cancers.* (2024) 16:2943. 10.3390/cancers16172943 39272800 PMC11394070

[129] Laine L Kaltenbach T Barkun A McQuaid K Subramanian V Soetikno R SCENIC international consensus statement on surveillance and management of dysplasia in inflammatory bowel disease. *Gastroenterology.* (2015) 148:639–51.e28. 10.1053/j.gastro.2015.01.031 25702852

[130] Gordon H Biancone L Fiorino G Katsanos K Kopylov U Al Sulais E ECCO guidelines on inflammatory bowel disease and malignancies. *J Crohns Colitis.* (2023) 17:827–54. 10.1093/ecco-jcc/jjac187 36528797

[131] Maeda Y Kudo S Ogata N Misawa M Mori Y Mori K Can artificial intelligence help to detect dysplasia in patients with ulcerative colitis? *Endoscopy.* (2021) 53:E273–4. 10.1055/a-1261-2944 33003217

[132] Fukunaga S Kusaba Y Ohuchi A Nagata T Mitsuyama K Tsuruta O Is artificial intelligence a superior diagnostician in ulcerative colitis? *Endoscopy.* (2021) 53:E75–6. 10.1055/a-1195-1986 32590852

[133] Mayo Clinic. *Gastroenterology and Hepatology. Development of an artificial intelligence tool for detecting colorectal lesions in inflammatory bowel disease.* (n.d.). Available online at: https://mayoclinic.elsevierpure.com/en/publications/development-of-an-artificial-intelligence-tool-for-detecting-colo/fingerprints (accessed July 19, 2025).

[134] Abdelrahim M Siggens K Iwadate Y Maeda N Htet H Bhandari P. New AI model for neoplasia detection and characterisation in inflammatory bowel disease. *Gut.* (2024) 73:725–8. 10.1136/gutjnl-2023-330718 38395438

[135] Yamamoto S Kinugasa H Hamada K Tomiya M Tanimoto T Ohto A The diagnostic ability to classify neoplasias occurring in inflammatory bowel disease by artificial intelligence and endoscopists: a pilot study. *J Gastroenterol Hepatol.* (2022) 37:1610–6. 10.1111/jgh.15904 35644932

[136] Xia J Xia T Pan J Gao F Wang S Qian Y Use of artificial intelligence for detection of gastric lesions by magnetically controlled capsule endoscopy. *Gastrointest Endosc.* (2021) 93:133–9.e4. 10.1016/j.gie.2020.05.027 32470426

[137] Dhali A Kipkorir V Maity R Srichawla B Biswas J Rathna R Artificial intelligence-assisted capsule endoscopy versus conventional capsule endoscopy for detection of small bowel lesions: a systematic review and meta-analysis. *J Gastroenterol Hepatol.* (2025) 40:1105–18. 10.1111/jgh.16931 40083189 PMC12062924

[138] Singh T Jha S Bhatt N. Machine learning methodologies in video capsule endoscopy-based bleeding analysis: a systematic review of progress and prospects (2008–2024). *Eng Appl Artif Intell.* (2025) 159:111659. 10.1016/j.engappai.2025.111659

[139] Nautiyal D Dhir M Singh T. Real-time, multi-task mobile application for automatic bleeding and non-bleeding frame analysis in video capsule endoscopy using an ensemble of faster R-CNN and LinkNet. *Int. J. Imaging Syst. Technol.* (2025) 35:e70171. 10.1002/ima.70171

[140] Handa P Goel N Indu S Gunjan D. A multi-label dataset and its evaluation for automated scoring system for cleanliness assessment in video capsule endoscopy. *Phys Eng Sci Med.* (2024) 47:1213–26. 10.1007/s13246-024-01441-w 38884670

[141] Mascarenhas Saraiva M Ferreira J Afonso J Mendes F Sonnier W Rosa B Real-life clinical validation of artificial intelligence-assisted detection and differentiation of pleomorphic lesions in capsule endoscopy. *Am J Gastroenterol.* (2025): 10.14309/ajg.0000000000003756 Online ahead of print. 40874973

[142] Oh D Hwang Y Kim S Nam J Jung M Lim Y. Reading of small bowel capsule endoscopy after frame reduction using an artificial intelligence algorithm. *BMC Gastroenterol.* (2024) 24:80. 10.1186/s12876-024-03156-4 38388860 PMC10885475

[143] Kwon Y Park T Kim S Park Y Lee J Lee S Deep learning-based localization and lesion detection in capsule endoscopy for patients with suspected small-bowel bleeding. *World J Gastroenterol.* (2025) 31:106819. 10.3748/wjg.v31.i27.106819 40741104 PMC12305051

[144] Dell’Anna G Mandarino F Centanni L Lodola I Fanizza J Fasulo E Transforming gastrointestinal diagnosis with molecular endoscopy: challenges and opportunities. *Int J Mol Sci.* (2025) 26:4834. 10.3390/ijms26104834 40429975 PMC12112569

[145] Zhang D Wu C Yang Z Yin H Liu Y Li W The application of artificial intelligence in EUS. *Endosc Ultrasound.* (2024) 13:65–75. 10.1097/eus.0000000000000053 38947752 PMC11213611

[146] Mahajan S Siyu S Bhutani M. What can artificial intelligence do for EUS? *Endosc Ultrasound.* (2025) 14:1–3. 10.1097/eus.0000000000000102 40151598 PMC11939944

[147] Takasu A Kogure H Dai Z Yamada Y Nakayama M Bechara R Impact of introducing artificial intelligence on colonoscopy: a retrospective study on potential benefits and drawbacks. *J Gastroenterol Hepatol.* (2025) 40:2258–66. 10.1111/jgh.17040 40598760

[148] Bretthauer M Ahmed J Antonelli G Beaumont H Beg S Benson A Use of computer-assisted detection (CADe) colonoscopy in colorectal cancer screening and surveillance: European society of gastrointestinal *Endoscopy* (ESGE) position statement. *Endoscopy.* (2025) 57:667–73. 10.1055/a-2543-0370 40148135

[149] Trovato A Turshudzhyan A Tadros M. Serrated lesions: a challenging enemy. *World J Gastroenterol.* (2021) 27:5625–9. 10.3748/wjg.v27.i34.5625 34629791 PMC8473594

[150] Ahmad O González-Bueno Puyal J Brandao P Kader R Abbasi F Hussein M Performance of artificial intelligence for detection of subtle and advanced colorectal neoplasia. *Dig Endosc.* (2022) 34:862–9. 10.1111/den.14187 34748665

[151] Hassan C Bisschops R Sharma P Mori Y. Colon cancer screening, surveillance, and treatment: novel artificial intelligence driving strategies in the management of colon lesions. *Gastroenterology.* (2025) 169:444–55. 10.1053/j.gastro.2025.02.021 40054749

[152] Coluccio C Jacques J Hritz I Boskoski I Abdelrahim M Bove V Simulators and training models for diagnostic and therapeutic gastrointestinal endoscopy: European society of gastrointestinal *Endoscopy* (ESGE) Technical and technology review. *Endoscopy.* (2025) 57:796–813. 10.1055/a-2569-7736 40185129

[153] Kücking F Hübner U Przysucha M Hannemann N Kutza J Moelleken M Automation bias in AI-Decision support: results from an empirical study. *Stud Health Technol Inform.* (2024) 317:298–304. 10.3233/SHTI240871 39234734

[154] Abdulnour R Gin B Boscardin C. Educational strategies for clinical supervision of artificial intelligence use. *N Engl J Med.* (2025) 393:786–97. 10.1056/NEJMra2503232 40834302

[155] Ahmad O. Endoscopist deskilling: an unintended consequence of AI-assisted colonoscopy? *Lancet Gastroenterol Hepatol.* (2025) 10:872–3. 10.1016/S2468-1253(25)00164-5 40816300

[156] Budzyń K Romańczyk M Kitala D Kołodziej P Bugajski M Adami H Endoscopist deskilling risk after exposure to artificial intelligence in colonoscopy: a multicentre, observational study. *Lancet Gastroenterol Hepatol.* (2025) 10:896–903. 10.1016/S2468-1253(25)00133-5 40816301

[157] ASGE AI Task Force, Parasa S Berzin T Leggett C Gross S Repici A Consensus statements on the current landscape of artificial intelligence applications in endoscopy, addressing roadblocks, and advancing artificial intelligence in gastroenterology. *Gastrointest Endosc.* (2025) 101:2–9.e1. 10.1016/j.gie.2023.12.003 38639679

[158] Brunyé T Mitroff S Elmore J. Artificial intelligence and computer-aided diagnosis in diagnostic decisions: 5 questions for medical informatics and human-computer interface research. *J Am Med Inform Assoc.* (2025): 10.1093/jamia/ocaf123 Online ahead of print. 41101774 PMC12844592

[159] Xu H Gong T Song X Chen Q Bao Q Yao W Artificial intelligence performance in image-based cancer identification: umbrella review of systematic reviews. *J Med Internet Res.* (2025) 27:e53567. 10.2196/53567 40167239 PMC12000792

[160] Palak, Mangotra H Goel N. Effect of selection bias on automatic colonoscopy polyp detection. *Biomed Signal Processing Control.* (2023) 85:104915. 10.1016/j.bspc.2023.104915

[161] Ali S Ghatwary N Jha D Isik-Polat E Polat G Yang C Assessing generalisability of deep learning-based polyp detection and segmentation methods through a computer vision challenge. *Sci Rep.* (2024) 14:2032. 10.1038/s41598-024-52063-x 38263232 PMC10805888

[162] Jong M Jaspers T Kusters C Jukema J Fockens KN Challenges in implementing endoscopic artificial intelligence: the impact of real-world imaging conditions on Barrett’s neoplasia detection. *United European Gastroenterol J.* (2025) 13:929–37. 10.1002/ueg2.12760 40116287 PMC12269737

[163] Bernal J Tajkbaksh N Sanchez F Matuszewski B Hao Chen Lequan Yu Comparative validation of polyp detection methods in video colonoscopy: results from the MICCAI 2015 endoscopic vision challenge. *IEEE Trans Med Imaging.* (2017) 36:1231–49. 10.1109/TMI.2017.2664042 28182555

[164] Jha D Ali S Hicks S Thambawita V Borgli H Smedsrud P A comprehensive analysis of classification methods in gastrointestinal endoscopy imaging. *Med Image Anal.* (2021) 70:102007. 10.1016/j.media.2021.102007 33740740

[165] Messmann H Bisschops R Antonelli G Libânio D Sinonquel P Abdelrahim M Expected value of artificial intelligence in gastrointestinal endoscopy: European society of gastrointestinal *Endoscopy* (ESGE) position statement. *Endoscopy.* (2022) 54:1211–31. 10.1055/a-1950-5694 36270318

